# Synthesis and Antiviral Evaluation of 3′-Fluoro-5′-norcarbocyclic Nucleoside Phosphonates Bearing Uracil and Cytosine as Potential Antiviral Agents

**DOI:** 10.3390/molecules25163708

**Published:** 2020-08-14

**Authors:** Pierre-Yves Geant, Jean-Pierre Uttaro, Christian Périgaud, Christophe Mathé

**Affiliations:** Institut des Biomolécules Max Mousseron (IBMM), UMR 5247, Université de Montpellier, CNRS, ENSCM, cc 1705, Site Triolet, Place Eugène Bataillon, 34095 Montpellier CEDEX 5, France; pygeant@yahoo.fr (P.-Y.G.); jean-pierre.uttaro@umontpellier.fr (J.-P.U.); christian.perigaud@umontpellier.fr (C.P.)

**Keywords:** nucleoside analogues, carbocyclic, phosphonates, antiviral

## Abstract

Carbocyclic nucleoside analogues are an essential class of antiviral agents and are commonly used in the treatment of viral diseases (hepatitis B, AIDS). Recently, we reported the racemic synthesis and the anti-human immunodeficiency virus activities (HIV) of 3′-fluoro-5′-norcarbocyclic nucleoside phosphonates bearing purines as heterocyclic base. Based on these results, the corresponding racemic norcarbocyclic nucleoside phosphonates bearing pyrimidine bases were synthesized. The prepared compounds were evaluated against HIV, but none of them showed marked antiviral activity compared to their purine counterparts.

## 1. Introduction

Nucleoside analogues have been an important part of the therapeutic arsenal used in the treatment of viral diseases for several years [1]. Among them, a class of modified nucleoside analogues are carbocyclic nucleosides, in which the endocyclic oxygen of the furanose ring of the natural nucleosides is replaced by an isosteric methylene group. The mode of action of these compounds involves their intracellular phosphorylation to their 5′-triphosphate form which can interact with virus-specific polymerases, acting as a competitive inhibitor or an alternate substrate for these target enzymes and finally preventing further viral nucleic acid chain elongation. 

**Figure 1:**
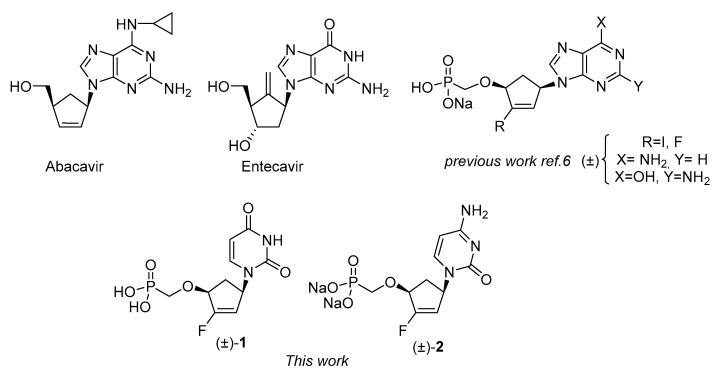
Example of antiviral carbocyclic nucleosides and structures of the target compounds.

As illustrated with the approval of carbocyclic nucleosides such as abacavir and entecavir as anti-HIV and anti-HBV drugs, respectively, the design and synthesis of novel carbocyclic scaffolds could lead to the discovery of new antiviral agents [2,3]. As a part of our ongoing research on 5′-norcarbocyclic nucleoside phosphonates [4,5] as potential anti-viral agents, we have described the synthesis and the anti-HIV activities of 3′-fluoro-5′-norcarbocyclic nucleoside phosphonates bearing purines as heterocyclic base (Figure 1) [6].

In such structures, the presence of a P-C bond instead of the hydrolysable P-O induces a metabolic stability of the linkage mimicking the phosphate moiety. Thus, these results prompted us to undertake the synthesis and the antiviral evaluation of the corresponding 3′-fluoro-4′-5′-norcarbocyclic nucleoside phosphonates bearing uracil (±)-**1** and cytosine (±)-**2**, respectively.

## 2. Results

### 2.1. Chemistry

**Scheme 1:**
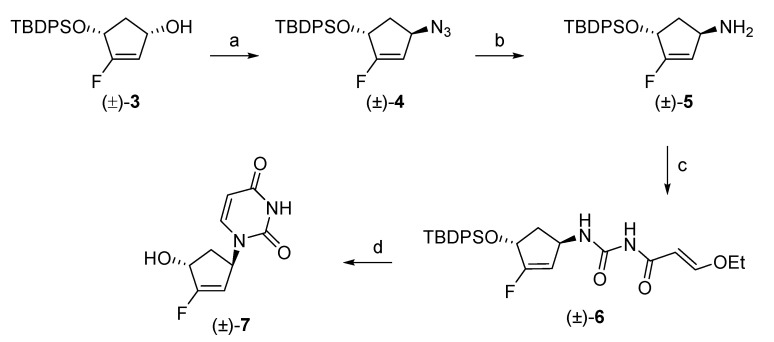
Reagents and conditions: (a) DPPA, DIAD, PPh_3_, THF/CH_2_Cl_2_, 0 °C, 30 min, 67%. (b) LiAlH_4_, THF, 0 °C to RT, 30 min, 84%. (c) (*E*)-3-ethoxyacryloyl chloride, AgOCN, toluene, reflux, 30 min; then (±)-**5**, DMF, −20 °C to RT, 5 h, 98%. (d) HCl (2N), EtOH, reflux, 12 h, 78%.

The strategy for the synthesis of the target carbocyclic nucleosides was based upon the preparation of compound (±)-**7** as a common precursor (Scheme 1). The latter was synthesized from alcohol (±)-**3** obtained according to our previously described procedure [6]. The azide with trans-configuration was obtained in good yield using a Mitsunobu reaction [7] in the presence of diphenylphosphorylazide (DPPA), triphenylphosphine and diisopropyl azodicarboxylate (DIAD). From azido derivative (±)-**4**, the required amine (±)-**5** was obtained after reduction with LiAlH_4_ in 84% yield. Finally, introduction of the uracil moiety of the common precursor (±)-**7** was achieved through a two steps procedure [8] with 76% yield by treatment of (±)-**5** with (*E*)-3-ethoxyacryloyl chloride followed by a cyclization reaction under acidic conditions.

**Scheme 2:**
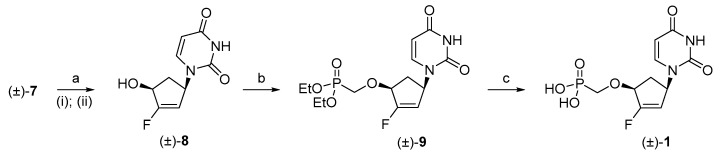
Reagents and conditions: (a) (i) BzOH, DIAD, PPh_3_, THF, RT, 1 h; (ii) K_2_CO_3_, MeOH, RT, 4 h, 93% over two steps. (b) (EtO)_2_P(O)CH_2_OTs, NaH, DMF, RT, 3 days. (c) TMSBr, DMF, RT, 24 h, 24% from (±)-**8**.

Synthesis of 3′-fluoro-5′-norcarbocyclic nucleoside phosphonates bearing uracil was carried out in four steps from compound (±)-**7** (Scheme 2). Briefly, the *trans*-(±)-**7** isomer was converted into the corresponding *cis*-isomer (±)-**8** in 93% yield using a Mitsunobu reaction in the presence of benzoic acid, triphenylphosphine and DIAD, then followed by the removal of the resulting benzoate group with potassium carbonate in methanol. This intermediate was transformed into the corresponding phosphonate (±)-**9** in the presence of sodium hydride and diethyl *p*-toluene sulfonyloxymethyl phosphonate [9]. Finally, the desired 3′-fluoro-5′-norcarbocyclic nucleoside phosphonate (±)-**1** bearing uracil was obtained after deprotection of the diethylphosphonoester group of the crude phosphonate (±)-**9** by treatment with trimethylsilyl bromide (TMSBr) in anhydrous DMF, followed by purification using reverse phase column chromatography.

**Scheme 3:**
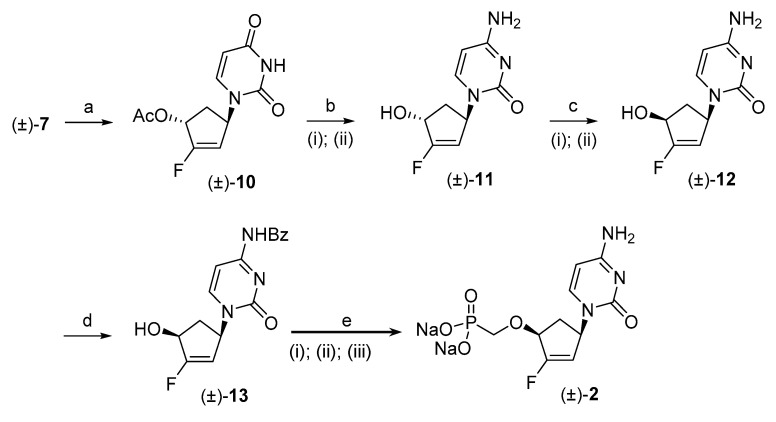
Reagents and conditions: (a) Ac_2_O, DMAP, NEt_3_, THF, RT, 1 h, 77%. (b) (i) Lawesson’s reagent, 1,2-dichloroethane, 83 °C, 20 h; (ii) methanolic ammonia, 100 °C, 4 h, 78% over two steps. (c) (i) BzOH, DIAD, PPh_3_, THF, RT, 2 h; (ii) K_2_CO_3_, MeOH, RT, 2 h, 60% over two steps. (d) Bz_2_O, DMF, RT, 24 h, 44%. (e) (i) (EtO)_2_P(O)CH_2_OTs, LiO*t*Bu, DMF, 40 °C, 1 h; (ii) TMSBr, DMF, RT, 24 h; (iii) methanolic ammonia, 50 °C, 4 h, 67% over three steps.

Then, we planned the synthesis of 3′-fluoro-5′-norcarbocyclic nucleoside phosphonate (±)-**2** bearing cytosine as nucleobase. The corresponding synthetic pathway is described in Scheme 3. In order to obtain the derivative (±)-**12**, the common precursor (±)-**7** was acetylated using the usual procedure (acetic anhydride, 4-dimethylaminopyridine and trimethylamine) to give compound (±)-**10**. The uracil moiety was transformed into cytosine nucleobase through a two-step procedure using Lawesson’s reagent followed by an amination of the nucleobase in the presence of methanolic ammonia at 100 °C. This treatment led to the concomitant hydrolysis of the acetyl group. The *trans* stereochemistry between the hydroxyl group and the nucleobase was inverted to afford (±)-**12** using the same protocol as previously described for alcohol (±)-**8**. From partially purified derivative (±)-**12**, the procedure for the synthesis of the desired 3′-fluoro-5′-norcarbocyclic nucleoside phosphonate (±)-**2** was slightly different to the one previously described for phosphonate (±)-**1**. A transient protection of the exocyclic amino function was performed by converting it in benzamide to avoid *N*-alkylation. Subsequently, *O*-alkylation of the protected nucleoside was achieved with LiO*t*Bu and diethyl *p*-toluene sulfonyloxymethyl phosphonate [9] followed by hydrolysis of the resulting diethyl phosphonate esters with TMSBr in anhydrous DMF. Finally, treatment of the deprotected phosphonate with methanolic ammonia at 50 °C followed by purification on reversed-phase column chromatography and ion exchange on a dowex resin provided the target 3′-fluoro-5′-norcarbocyclic nucleoside phosphonate (±)-**2** in 67% yield.

### 2.2. Antiviral Evaluation

The 3′-fluoro-5′-norcarbocyclic nucleoside phosphonate (±)-**1** and –(±)-**2** were evaluated for their antiviral activity in human peripheral blood mononuclear cells (PBMC) infected with HIV-1. None of them displayed antiviral activity (EC_50_ > 10 µM). No concomitant cytotoxicity for the target compounds was observed in PBM cells (CC_50_ > 10 µM). In contrast to the 3′-fluoro-5′-norcarbocyclic nucleoside phosphonates of the purine series, several factors could be responsible for the inactivity of these nucleoside derivatives. Their inability to enter cells or to serve as substrates for intracellular enzymes catalysing triphosphorylation, as well as a lack of inhibition of viral polymerase by their triphosphate forms, would all account for their inactivity against HIV.

## 3. Materials and Methods

### 3.1. General Information

Commercially available reagents were used as purchased without further purification, unless otherwise indicated. All solvents were freshly distilled under argon atmosphere: THF from a blue solution of sodium benzophenone ketyl radical, CH_2_Cl_2_, 1,2-dichloroethane and DMF from CaH_2_, pyridine and NEt_3_ from KOH and toluene from LiAlH_4_. All air and/or moisture sensitive reactions were carried out under an argon atmosphere and using standard syringe-cannula/septa techniques. ^1^H-, ^13^C-, ^31^P- and ^19^F-NMR spectra were recorded in CDCl_3_, MeOD or D_2_O solutions on DRX 400 (Bruker BioSpin Corporation, Billerica, MA, USA) or AM-300 spectrometers (Bruker, BioSpin Corporation, Billerica, MA, USA) and the chemicals shifts (*δ*) are reported in parts per millions calibrated using residual non deuterated solvents as internal references (CDCl_3_: 7.26 for ^1^H and 77.16 for ^13^C; MeOD: 3.31 for ^1^H and 49.0 for ^13^C; D_2_O: 4.79). ^19^F chemical shifts are reported using trichlorofluoromethane as external reference. For ^31^P-NMR spectra, chemical shifts are reported relative to external H_3_PO_4_. Signals are indicated as: s (singlet), d (doublet), t (triplet), q (quartet), m (multiplet) and br (broad). Analytical thin-layer chromatography was performed using Merck silica gel 60 F_254_ aluminium supported TLC plates (Art. 5554, Merck, Analtech, Newark, DE, USA); spots were visualized using a UV light and ethanolic acidic *p*-anisaldehyde solution or 5% ethanolic sulfuric acid solution, followed by heating. Purification by column chromatography was performed with silica gel 60 (230–400 mesh) or with reversed phase silica gel (RP-18, 25–40 µm). Ion exchange chromatography was performed on DOWEX 50WX8-200 resins (Na^+^ form) (Aldrich, Milwaukee, WI, USA). Mass spectra were recorded on a Q-TOF mass spectrometer using electrospray ionization (Micromass, Waters Corporation, Milford, MA, USA). HRMS were obtained with a Synapt G2S spectrometer (Waters Corporation, Milford, MA, USA) equipped with positive or negative electrospray source ionization (ESI), using Leu-enkephalin as an internal standard. The capillary voltage was set to 1.2 kV and the sampling cone voltage was set to 30 V. UV spectra were recorded with an Uvikon 931 (Kontron, El Cajon, CA, USA) spectrophotometer, *λ* are expressed in nm and ε in L.mol^−1^.cm^−1^. ^1^H-, ^13^C-, ^31^P- and ^19^F-NMR spectra for the synthesized compounds are available online in Supplementary Materials.

### 3.2. Syntheses

*((4-Azido-2-fluorocyclopent-2-en-1-yl)oxy)(t-butyl)diphenylsilane (±)-**4**.* To a stirred mixture of (±)-**3** (1.15 g, 3.22 mmol) and PPh_3_ (2.52 g, 9.61 mmol) in anhydrous THF/CH_2_Cl_2_ (1/1 *v/v*, 60 mL) at 0 °C were added, under an argon atmosphere, diphenylphosphoryl azide (DPPA, 2 mL, 9.31 mmol) and diisopropyl azodicarboxylate (DIAD, 1.8 mL, 9.14 mmol). The reaction mixture was stirred at 0 °C for 30 min then concentrated under reduced pressure. The residue was purified by flash chromatography on silica gel (petroleum ether/CH_2_Cl_2_ 100/0 to 85/15), affording (±)-**4** (825 mg, 67% yield) as a yellowish oil. Due to its instability (±)-**4** was quickly used for the next step. Rf = 0.75 (petroleum ether/EtOAc, 7/3). ^1^H-NMR (CDCl_3_, 300 MHz): *δ* 7.69–7.65 (m, 4H, ArH), 7.48–7.37 (m, 6H, ArH), 5.29 (dd, *J* = 2.5, 0.5 Hz, 1H, H3), 4.91–4.86 (m, 1H, H1), 4.49–4.43 (m, 1H, H4), 2.20 (dddd, *J* = 14.4, 7.6, 3.9, 0.9 Hz, 1H, H5a), 1.97 (dddd, *J* = 14.4, 7.2, 2.5, 1.1 Hz, 1H, H5b), 1.08 (s, 9H, 3 × CH_3_).

*4-((tert-Butyldimethylsilyl)oxy)-3-fluorocyclopent-2-enamine (±)-**5***. To a stirred solution of (±)-**4** (825 mg, 2.16 mmol) in anhydrous THF (70 mL) at 0 °C was added dropwise, under an argon atmosphere, LiAlH_4_ (1 M in THF, 3.3 mL, 3.3 mmol). The reaction mixture was allowed to warm to room temperature and stirred for additional 30 min. THF/H_2_O (15 mL, 9/1 *v/v*) were added dropwise then the mixture was filtrated through a short pad of celite^®^ and silica gel which was washed with MeOH. The filtrate was concentrated under reduced pressure to afford (±)-**5** (646 mg, 84% yield) as a colorless oil. Rf = 0.3 (CH_2_Cl_2_/MeOH, 9/1). ^1^H-NMR (CDCl_3_, 400 MHz): *δ* 7.69–7.65 (m, 4H, ArH), 7.45–7.35 (m, 6H, ArH), 5.21 (d, *J* = 2.1 Hz, 1H, H2), 4.87–4.83 (m, 1H, H4), 4.07–4.02 (m, 1H, H1), 2.25–2.19 (m, 1H, H5a), 1.69–1.61 (ddd, *J* = 14.0, 7.3, 3.4 Hz, 1H, H5b), 1.40 (br s, 1H, NH_2_), 1.07 (s, 9H, 3 × CH_3_). ^13^C-NMR (CDCl_3_, 100 MHz): *δ* 163.5 (d, *J* = 287 Hz, C3), 136.0 (C_Ar_), 135.9 (C_Ar_), 134.0 (Cq), 133.5 (Cq), 130.0 (C_Ar_), 129.9 (C_Ar_), 127.9 (C_Ar_), 127.7 (C_Ar_), 111.3 (d, *J* = 6.8 Hz, C2), 72.7 (d, *J* = 21.5 Hz, C4), 50.7 (d, *J* = 9.8 Hz, C1), 43.7 (d, *J* = 5.6 Hz, C5), 27.0 (3 × CH_3_), 19.3 (Cq). ^19^F-NMR (CDCl_3_, 376 MHz): *δ* −127.2.

*(E)-N-((4-((tert-Butyldiphenylsilyl)oxy)-3-fluorocyclopent-2-en-1-yl)carbamoyl)-3-ethoxyacrylamide (±)-**6***. (E)-3-ethoxyacryloyl chloride (280 mg, 2.08 mmol) was added to a suspension of AgOCN (465 mg, 3.12 mmol) in anhydrous toluene (4 mL) under an argon atmosphere. The mixture was refluxed for 30 min then cooled to room temperature and filtrated through a pad of celite^®^. The filtrate was added dropwise at −20 °C to a solution of amine (±)-**5** (184 mg, 0.52 mmol) in anhydrous DMF (7 mL) under an argon atmosphere. The reaction mixture was allowed to reach room temperature and stirred for 5 h. The solvents were evaporated under reduced pressure and the residue was purified by flash chromatography on silica gel (petroleum ether/EtOAc, 100/0 to 60/40), affording (±)-**6** (252 mg, 98% yield) as a colorless oil. Rf = 0.24 (petroleum ether/EtOAc, 7/3). ^1^H-NMR (CDCl_3_, 400 MHz): *δ* 9.24 (s, 1H, NH), 8.53 (d, *J* = 7.7 Hz, 1H, NH), 7.68–7.64 (m, 4H, ArH), 7.58 (d, *J* = 12.2 Hz, 1H, H3), 7.45–7.35 (m, 6H, ArH), 5.28–5.25 (m, 2H, H2 and H2′), 4.98–4.93 (m, 1H, H1′), 4.87–4.85 (m, 1H, H4′), 3.93 (q, *J* = 7.1 Hz, 2H, CH_2_-CH_3_), 2.30 (ddd, *J* = 14, 7.9, 2.9 Hz, 1H, H5′a), 1.88 (ddd, *J* = 14, 7.3, 3.3 Hz, 1H, H5′b), 1.33 (t, *J* = 7.1 Hz, 3H, CH_2_-CH_3_), 1.07 (s, 9H, 3 × CH_3_). ^13^C-NMR (CDCl_3_, 100 MHz): *δ* 168.0 (CO), 164.6 (d, *J* = 287 Hz, C3′), 163.0 (C3), 154.6 (CO), 135.9 (C_Ar_), 135.7 (C_Ar_), 133.6 (Cq), 133.1 (Cq), 129.8 (2 × C_Ar_), 127.7 (C_Ar_), 127.6 (C_Ar_), 107.1 (d, *J* = 10.2 Hz, C2′), 97.8 (C2), 71.9 (d, *J* = 21.1 Hz, C4′), 67.5 (CH_2_-CH_3_), 49.0 (d, *J* = 11.5 Hz, C1′), 40.8 (d, *J* = 5.1 Hz, H5′), 26.8 (3 × CH_3_), 19.2 (Cq), 14.4 (CH_2_-CH_3_). ^19^F-NMR (CDCl_3_, 376 MHz): *δ* −124.4. HRMS (ESI^−^): calculated for C_27_H_32_FN_2_O_4_Si [M − H]^−^ 495.2115; found: 495.2116.

*Trans-1-(3-fluoro-4-hydroxycyclopent-2-en-1-yl)pyrimidine-2,4(1H,3H)-dione (±)-**7***. Hydrochloric acid (2N aqueous solution, 3.9 mL, 7.33 mmol) was added to a stirred solution of (±)-**6** (1.2 g, 2.43 mmol) in EtOH (35 mL). The reaction mixture was refluxed overnight, then cooled to room temperature and concentrated under reduced pressure. The crude material was purified by flash chromatography on silica gel (CH_2_Cl_2_/MeOH, 100/0 to 90/10) to give (±)-**7** (401 mg, 78% yield) as a white foam. Rf = 0.34 (CH_2_Cl_2_/MeOH, 9/1). ^1^H-NMR (CD_3_OD, 400 MHz): *δ* 7.48 (d, *J* = 8.0 Hz, 1H, H6), 5.67 (d, *J* = 8.0 Hz, 1H, H5), 5.68–5.62 (m, 1H, H1′), 5.29 (d, *J* = 2.2 Hz, 1H, H2′), 4.90–4.86 (m, 1H, H4′), 2.34 (dddd, *J* = 14.8, 8.2, 3.2, 1.3 Hz, 1H, H5′a), 2.19 (ddd, *J* = 14.8, 7.5, 3.6 Hz, 1H, H5′b). ^13^C-NMR (CD_3_OD, 100 MHz): *δ* 168.5 (d, *J* = 288 Hz, C3′), 166.4 (CO), 152.8 (CO), 143.2 (C6), 105.8 (d, *J* = 13.0 Hz, C2′), 102.8 (C5), 71.1 (d, *J* = 21.4 Hz, C4′), 57.5 (d, *J* = 12.0 Hz, C1′), 40.1 (d, *J* = 5.7 Hz, C5′). ^19^F-NMR (CD_3_OD, 376 MHz): *δ* −123.4. UV (EtOH 95) λ_max_ = 265 nm (ε_max_ = 9800). HRMS (ESI^−^): calculated for C_9_H_8_FN_2_O_3_ [M − H]^−^ 211.0519; found: 211.0519.

*Cis-1-(3-fluoro-4-hydroxycyclopent-2-en-1-yl)pyrimidine-2,4(1H,3H)-dione (±)-**8***. Diisopropyl azodicarboxylate (DIAD, 1.67 mL, 8.51 mmol) was added dropwise, under an argon atmosphere, to a stirred mixture of (±)-**7** (361 mg, 1.7 mmol), PPh_3_ (2.23 g, 8.51 mmol) and benzoic acid (1.09 g, 8.51 mmol) in anhydrous THF (37 mL) at 0 °C. The mixture was allowed to reach room temperature and stirred for 1 h, then the solvents were evaporated under reduced pressure. The residue was dissolved in MeOH (37 mL), K_2_CO_3_ (588 mg, 4.25 mmol) was added and the solution was stirred at room temperature for 4 h. The solvents were evaporated under reduced pressure and the crude material was purified by flash chromatography on silica gel (CH_2_Cl_2_/MeOH, 93/7) yielding (±)-**8** (336 mg, 93%) as a white solid. Rf = 0.39 (CH_2_Cl_2_/MeOH, 9/1). ^1^H-NMR (D_2_O, 400 MHz): *δ* 7.70 (d, *J* = 8.0 Hz, 1H, H6), 5.89 (d, *J* = 8.0 Hz, 1H, H5), 5.44–5.39 (m, 2H, H2′ and H1′), 4.78–4.76 (m, 1H, H4′), 3.11–3.03 (m, 1H, H5′a), 1.67–1.61 (m, 1H, H5′b). ^13^C-NMR (D_2_O, 100 MHz): *δ* 166.5 (CO), 165.2 (d, *J* = 286 Hz, C3′), 152.2 (CO), 143.3 (C6), 105.0 (d, *J* = 13.1 Hz, C2′), 102.1 (C5), 68.6 (d, *J* = 21.4, C4′), 54.0 (d, *J* = 11.5 Hz, C1′), 37.8 (d, *J* = 5.6 Hz, C5′). ^19^F-NMR (D_2_O, 376 MHz): *δ* −123.1. UV (EtOH 95) λ_max_ = 266 nm (ε_max_ = 6400). HRMS (ESI^−^): calculated for C_9_H_8_FN_2_O_3_ [M − H]^−^ 211.0519; found: 211.0520.

*((4-(2,4-Dioxo-3,4-dihydropyrimidin-1(2H)-yl)-2-fluorocyclopent-2-en-1-yl)oxy)methylphosphonic acid (±)-**1***. A solution of (±)-**8** (26 mg, 0.123 mmol) in anhydrous DMF (0.5 mL) was added, under an argon atmosphere, to a suspension of NaH (60% in mineral oil, 25 mg, 0.625 mmol) in anhydrous DMF (0.5 mL). The mixture was stirred at room temperature for 15 min then a solution of diethyl-(tosyloxymethyl)phosphonate (47 mg, 0.146 mmol) was added. The reaction mixture was stirred at room temperature for 3 days, then AcOH (50 μL) was added and the solvents were evaporated under reduced pressure to furnish crude (±)-**9**. This intermediate was dissolved in anhydrous DMF (1.5 mL) and TMSBr (80 μL, 0.617 mmol) was added dropwise at 0 °C under an argon atmosphere. The solution was stirred at room temperature for 16 h until a further aliquot of TMSBr was added (80 μL, 0.617 mmol). After 4 additional hours of stirring, TMSBr (160 μL, 1.3 mmol) was again added and the mixture was stirred for a further 4 h at room temperature then, the reaction was stopped by adding triethylammonium bicarbonate buffer (TEAB 1M, pH 7) and concentrated to dryness under high vacuum. Purification by reverse-phase chromatography on RP-18 (H_2_O/MeOH, 100/0 to 80/20) followed by column chromatography on silica gel (*i*-PrOH/NH_4_OH/H_2_O, 7/2/1) afforded the phosphonate (±)-**1** (9 mg, 24% yield). Rf = 0.15 (*i*-PrOH/NH_4_OH/H_2_O, 7/2/1). ^1^H-NMR (D_2_O, 400 MHz): *δ* 7.77 (d, *J* = 8.0 Hz, 1H, H6), 5.89 (d, *J* = 8.0 Hz, 1H, H5), 5.51–5.40 (m, 2H, H1′ and H3′), 4.63 (dd, *J* = 7.4, 2.8 Hz, 1H, H4′), 3.71 (d, *J* = 9.2 Hz, 2H, CH_2_P), 3.00 (ddd, *J* = 15.9, 7.9, 7.9 Hz, 1H, H5′a), 1.97–1.73 (m, 1H, H5′b). ^13^C-NMR (D_2_O, 100 MHz): *δ* 166.5 (CO), 164.5 (d, *J* = 286 Hz, C2′), 152.2 (CO), 143.7 (C6), 106.7 (d, *J* = 12.4 Hz, C3′), 102.2 (C5), 78.5 (C1′), 65.2 (CH2P), 53.8 (d, *J* = 11.4 Hz, C4′), 34.9 (C5′). ^19^F NMR (D_2_O, 376 MHz): *δ* −120.7. ^31^P-NMR (162 MHz, D_2_O): *δ* 14.7. UV (H_2_O) λ_max_ = 264 nm (ε_max_ = 8600). HRMS (ESI^−^): calculated for C_10_H_11_FN_2_O_6_P [M − H]^−^ 305.0339; found: 305.0341.

*Trans-4-(2,4-dioxo-3,4-dihydropyrimidin-1(2H)-yl)-2-fluorocyclopent-2-en-1-yl acetate (±)-**10***. To a stirred solution of (±)-**7** (370 mg, 1.75 mmol) in anhydrous THF (7 mL) were added NEt_3_ (0.37 mL, 2.65 mmol), DMAP (10 mg, 0.08 mmol) and Ac_2_O (0.2 mL, 2.12 mmol) under an argon atmosphere. The mixture was stirred at room temperature for 1 hour before concentration to dryness. The residue was dissolved in water (50 mL) and extracted with EtOAc (3 × 25 mL). The combined organic layers were washed with brine (50 mL), dried (MgSO_4_) and concentrated *in vacuo* to afford (±)-**10** (340 mg, 77%) as a white powder. Rf = 0.64 (CH_2_Cl_2_/MeOH, 9/1). ^1^H-NMR (MeOD, 400 MHz): *δ* 7.52 (d, *J* = 8.0 Hz, 1H, H6), 5.90–5.87 (m, 1H, H4′), 5.68 (d, *J* = 8 Hz, 1H, H5), 566–5.61 (m, 1H, H1′), 5.61 (d, *J* = 2.3 Hz, 1H, H3′), 2.48–2.41 (m, H5′a), 2.36 (ddd, *J* = 15.2, 7.6, 3.9 Hz, 1H, H5′b), 2.09 (s, 3H, CH_3_). ^13^C- NMR (MeOD, 100 MHz): *δ* 172.0 (C=O), 166.3 (C=O), 164.3 (d, *J* = 285.9 Hz, C3′), 152.6 (C=O), 143.4 (C6), 109.5 (d, *J* = 12.3 Hz, C2′), 102.9 (C5), 74.1 (d, *J* = 21.5 Hz, C4′), 57.7 (d, *J* = 11.4 Hz, C1′), 37.5 (d, *J* = 4.3 Hz, C5′), 20.7 (CH_3_). ^19^F-NMR (MeOD, 376 MHz): *δ* −123.3. UV (EtOH) λ_max_ = 265 (ε_max_ = 7600). HRMS (ESI^+^): calculated for C_11_H_12_N_2_O_4_F [M + H]^+^ 255.0781; Found 255.0780.

*Trans-4-amino-1-(3-fluoro-4-hydroxycyclopent-2-en-1-yl)pyrimidin-2(1H)-one (±)-**11***. Lawesson’s reagent (870 mg, 2.15 mmol) was added to a suspension of (±)-**10** (340 mg, 1.34 mmol) in anhydrous freshly distilled 1,2-dichloroethane (65 mL), at room temperature under an argon atmosphere. The mixture was refluxed for 20 h, then cooled to room temperature, concentrated and the residue was filtrated through a pad of silica gel (CH_2_Cl_2_/MeOH, 9/1). The filtrate was concentrated to vacuum and the resulting crude material was dissolved in NH_3_/MeOH (7N, 25 mL) before being heated in a Parr high pressure reactor at 100 °C for 4 h. Concentration to dryness and purification by column chromatography on silica gel (CH_2_Cl_2_/MeOH, 9/1) afforded (±)-**11** (220 mg, 78%) as a colorless oil. Rf = 0.33 (CH_2_Cl_2_/MeOH, 8/2). ^1^H-NMR (MeOD, 400 MHz): *δ* 7.49 (d, *J* = 7.4 Hz, 1H, H6), 5.88 (d, *J* = 7.4 Hz, 1H, H5), 5.72–5.66 (m, 1H, H1′), 5.29 (d, *J* = 2.4 Hz, 1H, H2′), H4′ signal in H_2_O peak, 2.38 (dddd, *J* = 14.6, 8.1, 3.2, 1.2 Hz, 1H, H5′a), 2.13 (ddd, *J* = 14.7, 7.7, 3.6 Hz, 1H, H5′b). ^13^C-NMR (MeOD, 100 MHz): *δ* 168.2 (d, *J* = 287.7 Hz, C3′), 167.5 (CN), 158.9 (CO), 143.2 (C6), 106.2 (d, *J* = 12.5 Hz, C2′), 96.3 (C5), 71.1 (d, *J* = 21.5 Hz, C4′), 58.1 (d, *J* = 11.7 Hz, C1′), 40.7 (d, *J* = 5.6 Hz, C5′). ^19^F-NMR (MeOD, 376 MHz): *δ* −123.78. UV (EtOH) λ_max_ = 276 nm (ε_max_ = 6400). HRMS (ESI^−^): calculated for C_9_H_11_N_3_O_2_F [M + H]^+^ 212.0835; Found 212.0837.

*Cis-4-amino-1-(3-fluoro-4-hydroxycyclopent-2-en-1-yl)pyrimidin-2(1H)-one (±)-**12***. Diisopropyl azodicarboxylate (DIAD, 0.98 mL, 5.00 mmol) was added dropwise to a stirred solution of (±)-**11** (210 mg, 1.00 mmol), PPh_3_ (1.31 g, 5.00 mmol) and benzoic acid (610 mg, 5.00 mmol) in anhydrous THF (20 mL) at 0 °C under an argon atmosphere. The mixture was allowed to reach room temperature and stirred for 2 h before concentration to dryness under reduced pressure. The residue was dissolved in MeOH (20 mL) before K_2_CO_3_ (276 mg, 2.00 mmol) was added. The mixture was stirred at room temperature for 2 h then the solvents were evaporated under reduced pressure. Purification by flash chromatography on silica gel (CH_2_Cl_2_/MeOH, 9/1) afforded partially purified (±)-**12** (125 mg, 60%) as a colourless oil. Compound (±)-**12** was used in the next step without further purification. Rf = 0.23 (CH_2_Cl_2_/MeOH, 8/2). ^1^H-NMR (MeOD, 400 MHz): *δ* 7.65 (d, *J* = 7.4 Hz, 1H, H6), 5.91 (d, *J* = 7.4 Hz, 1H, H5), 5.52–5.47 (m, 1H, H1′), 5.29 (d, *J* = 2.6 Hz, 1H, H2′), 4.64 (dd, *J* = 7.8, 3.5 Hz, 1H, H4′), 4.45 (br s, 1H, NH), 3.04–2.96 (m, 1H, H5′a), 1.53 (ddd, *J* = 14.7, 6.0, 3.5 Hz, 1H, H5′b). ^13^C-NMR (MeOD, 100 MHz): *δ* 167.8 (d, *J* = 286.6 Hz, C3′), 167.5 (CN), 158.9 (CO), 143.6 (C6), 106.2 (d, *J* = 12.2 Hz, C2′), 96.5 (C5), 70.1 (d, *J* = 21.7 Hz, C4′), 55.5 (d, *J* = 10.8 Hz, C1′), 40.3 (d, *J* = 5.7 Hz, C5′). ^19^F-NMR (MeOD, 376 MHz): *δ* −124.87. UV (EtOH) λ_max_ = 275nm (ε_max_ = 4300). HRMS (ESI^+^): calculated for C_9_H_11_N_3_O_2_F [M + H]^+^ 212.0835; Found 212.0836.

*N-(1-(3-Fluoro-4-hydroxycyclopent-2-en-1-yl)-2-oxo-1,2-dihydropyrimidin-4-yl)benzamide (±)-**13***. To a stirred solution of (±)-**12** (125 mg, 0.592 mmol) in anhydrous DMF (2.5 mL) was added benzoic anhydride (127 mg, 0.561 mmol) under an argon atmosphere. The reaction mixture was stirred at room temperature for 24 h and then concentrated to dryness. The solid residue was washed with Et_2_O, then purified by flash chromatography on silica gel (CH_2_Cl_2_ to CH_2_Cl_2_/MeOH, 9/1) to afford (±)-**13** (81 mg, 44%) as a white solid. Rf = 0.46 (CH_2_Cl_2_/MeOH, 9/1). ^1^H-NMR (MeOD, 400 MHz): *δ* 8.11 (d, *J* = 7.4 Hz, 1H, CH_Ar_), 7.98–7.95 (m, 2H, CH_Ar_ and H6), 7.64–7.60 (m, 2H, CH_Ar_), 7.55–7.50 (m, 2H, CH_Ar_ and H5), 5.58 (ddd, *J* = 8.8, 6.1, 3.1 Hz, 1H, H1′), 5.34 (d, *J* = 2.6 Hz, 1H, H2′), 4.67 (dd, *J* = 7.8, 3.3 Hz, 1H, H4′), 3.08 (ddd, *J* = 14.8, 8.1, 8.1 Hz, 1H, H5′a), 1.64 (ddd, *J* = 14.9, 5.8, 3.3 Hz, 1H, H5′b). ^13^C-NMR (MeOD, 100 MHz): *δ* 168.1 (d, *J* = 287.8 Hz, C3′), 164.1 (CN), 158.2 (CO), 147.2 (C6), 134.2 (C_Ar_), 133.9 (CH_Ar_), 129.5 (CH_Ar_), 128.9 (CH_Ar_), 105.4 (d, *J* = 13.2 Hz, C2′), 99.1 (C5), 69.8 (d, *J* = 21.7 Hz, C4′), 56.6 (d, *J* = 10.8 Hz, C1′), 40.0 (d, *J* = 5.8 Hz, C5′). ^19^F-NMR (MeOD, 376 MHz): *δ* −122.4. UV (EtOH) λ_max_ = 260 nm (ε_max_ = 16900). HRMS (ESI^+^): calculated for C_16_H_15_N_3_O_3_F [M + H]^+^ 316.1097; Found 316.1097.

*Sodium-(((4-(4-amino-2-oxopyrimidin-1(2H)-yl)-2-fluorocyclopent-2-en-1-yl)oxy)methyl) phosphonate (±)-**2**.* (±)-**13** (78 mg, 0.25 mmol) and diethyl-(tosyloxymethyl)phosphonate (321 mg, 0.80 mmol) were dissolved in anhydrous DMF under an argon atmosphere and the solution was heated at 40 °C for 30 min. LiO*t*Bu (2.2M in THF, 0.27 mL, 0.59 mmol) was added and the reaction mixture was heated at 40 °C for a further 30 min. Concentration to dryness and purification by flash chromatography afforded the desired intermediate phosphonate contaminated with tosylate. This mixture was used in the next step without further purification. To a solution of intermediate phosphonate in anhydrous DMF (4 mL) at 0 °C and under an argon atmosphere was added dropwise TMSBr (0.32 mL, 2.5 mmol). The solution was stirred at room temperature for 24 h, then neutralized by adding triethylammonium bicarbonate buffer (TEAB 1 M, pH 7) and concentrated to dryness under high vacuum. The residue was dissolved in NH_3_/MeOH (7 M) and heated in a Parr high pressure reactor at 50 °C for 4 h. After concentration to dryness, purification by reverse-phase chromatography on RP-18 (H_2_O to H_2_O/MeOH, 50/50) followed by an ion exchange on DOWEX 50WX2 (Na^+^ form) afforded (±)-**2** (59 mg, 67%) as a white powder. ^1^H-NMR (D_2_O, 400 MHz): *δ* 7.74 (d, *J* = 7.4 Hz, 1H, H6), 6.06 (d, *J* = 7.4 Hz, 1H, H5), 5.49–5.45 (m, 2H, H3′ and H4′), 4.64–4.61 (m, 1H, H1′), 3.68 (d, *J* = 9.4 Hz, 2H, OCH_2_P), 3.03–2.95 (m, 1H, H5′a), 1.81–1.77 (m, 1H, H5′b). ^13^C-NMR (D_2_O, 100 MHz): *δ* 165.9 (CN), 164.2 (d, *J* = 286.2 Hz, C2′), 158.2 (CO), 143.3 (C6), 107.2 (d, *J* = 12.1 Hz, C3′), 96.4 (C5), 78.3 (d, *J* = 20.4 Hz, C1′), 65.8 (d, *J* = 155.6 Hz, CH_2_P), 54.3 (d, *J* = 11.0 Hz, C4′), 35.3 (d, *J* = 5.3 Hz, C5′). ^19^F-NMR (D_2_O, 376 MHz): *δ* −121.35. ^31^P-NMR (D_2_O, 162 MHz): *δ* 14.6. UV (H_2_O) λ_max_ = 274 nm (ε_max_ = 6500). HRMS (ESI^−^): calculated for C_10_H_14_FN_3_O_5_P [M + H]^+^ 306.0655; Found 306.0656.

### 3.3. Antiviral Activity

Peripheral blood mononuclear cells (PBMC) from healthy donors were isolated and stimulated for 3 days with 1 µg/mL of phytohemagglutinin-P (PHA-P, Sigma) and 5 IU/mL of recombinant human interleukin-2 (rHuIL-2, Roche, Florham Park, NJ, USA). PBMC were growth under CO_2_ in a humid atmosphere at 37 °C in RPMI-1640 glutamax medium supplemented with antibiotics (penicillin, streptomycin, neomycin), 10% fetal calf serum (FCS, previously inactivated by heat) and 10 UI/mL of rHuIL-2. The HIV-1 LAI strain was previously described [10]. PHA-P activated PBMCs (1.5 × 10^5^ cells) were pre-treated for 30 min by increasing concentrations of the various compounds to be tested and then infected with 100% infectious tissue culture doses 50% (TCID_50_) of the HIV-1-LAI. Supernatants were collected at day 7 post infection and stored at −20 °C. Viral replication was measured by quantifying reverse transcriptase activity in cell culture supernatants by the use of Lenti kit RT (Cavidi, Uppsala, Sweden). Cytotoxicity of the compounds was evaluated in uninfected PHA-P PBMC by MTT assay (Promega, Madison, WI, USA) on day 7. Experiments were performed in triplicate and repeated with another blood donors. Data analyses were performed using SoftMax^®^ Pro 4.6 sofware (Molecular Devices, San Jose, CA, USA). Percent of inhibition of RT activity or cell viability were plotted *versus* compound concentration and fitted with quadratic curves to determine 50% effective concentration (EC_50_) and 50% cytotoxic concentration (CC_50_).

## 4. Patents

C. Mathé, C. Périgaud, J.-P. Uttaro, P.-Y. Geant (Université de Montpellier, CNRS), Int. PCT Pub. No. WO2017109388, 2017.

## Supplementary Materials

The following data are available online. ^1^H-, ^13^C-, ^31^P- and ^19^F-NMR spectra for the synthesized compounds.
