# Supplementary material for: Synthesis and Antiviral Evaluation of 3′-Fluoro-5′-norcarbocyclic Nucleoside Phosphonates Bearing Uracil and Cytosine as Potential Antiviral Agents
Source: Molecules. 2020 Aug 14;25(16):3708. doi: 10.3390/molecules25163708 (PMC7466164; doi:10.3390/molecules25163708)
Supplement: Supplementary file 1 [file molecules-25-03708-s001.pdf]

Supporting Information

Synthesis and antiviral evaluation of 3′-fluoro-5′-norcarbocyclic nucleoside phosphonates bearing uracil and cytosine as potential antiviral agents

Pierre-Yves Geant, Jean-Pierre Uttaro, Christian Périgaud and Christophe Mathé*

Institut des Biomolécules Max Mousseron (IBMM), UMR 5247, Université de Montpellier, CNRS, ENSCM, cc 1705, Site Triolet, Place Eugène Bataillon, 34095 Montpellier cedex 5 (France) ; pygeant@yahoo.fr; jean-pierre.uttaro@umontpellier.fr; christian.perigaud@umontpellier.fr;

* Correspondence: christophe.mathe@umontpellier.fr

Table of Contents:

^{1}H, ^{13}C, ^{31}P and ^{19}F NMR spectra for all compounds S2-S34

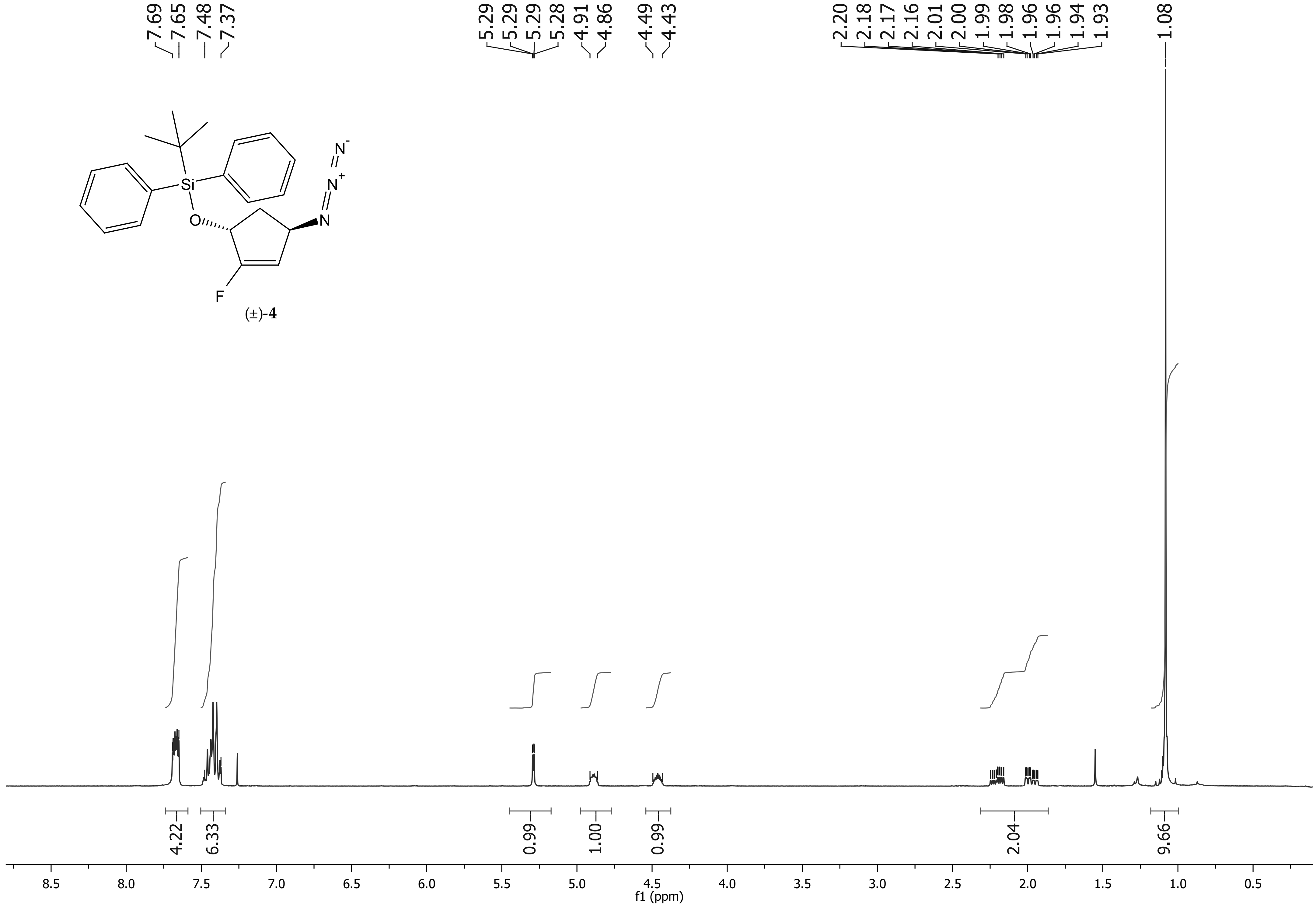
7.69
7.65
7.48
7.37
5.29
5.29
5.29
5.28
4.91
4.86
4.49
4.43
2.20
2.18
2.17
2.16
2.01
2.00
1.99
1.98
1.96
1.96
1.94
1.93
1.08
Si
O
N
N+
N-
F
(±)-4
4.22
6.33
0.99
1.00
0.99
2.04
9.66
8.5
8.0
7.5
7.0
6.5
6.0
5.5
5.0
4.5
4.0
3.5
3.0
2.5
2.0
1.5
1.0
0.5
f1 (ppm)

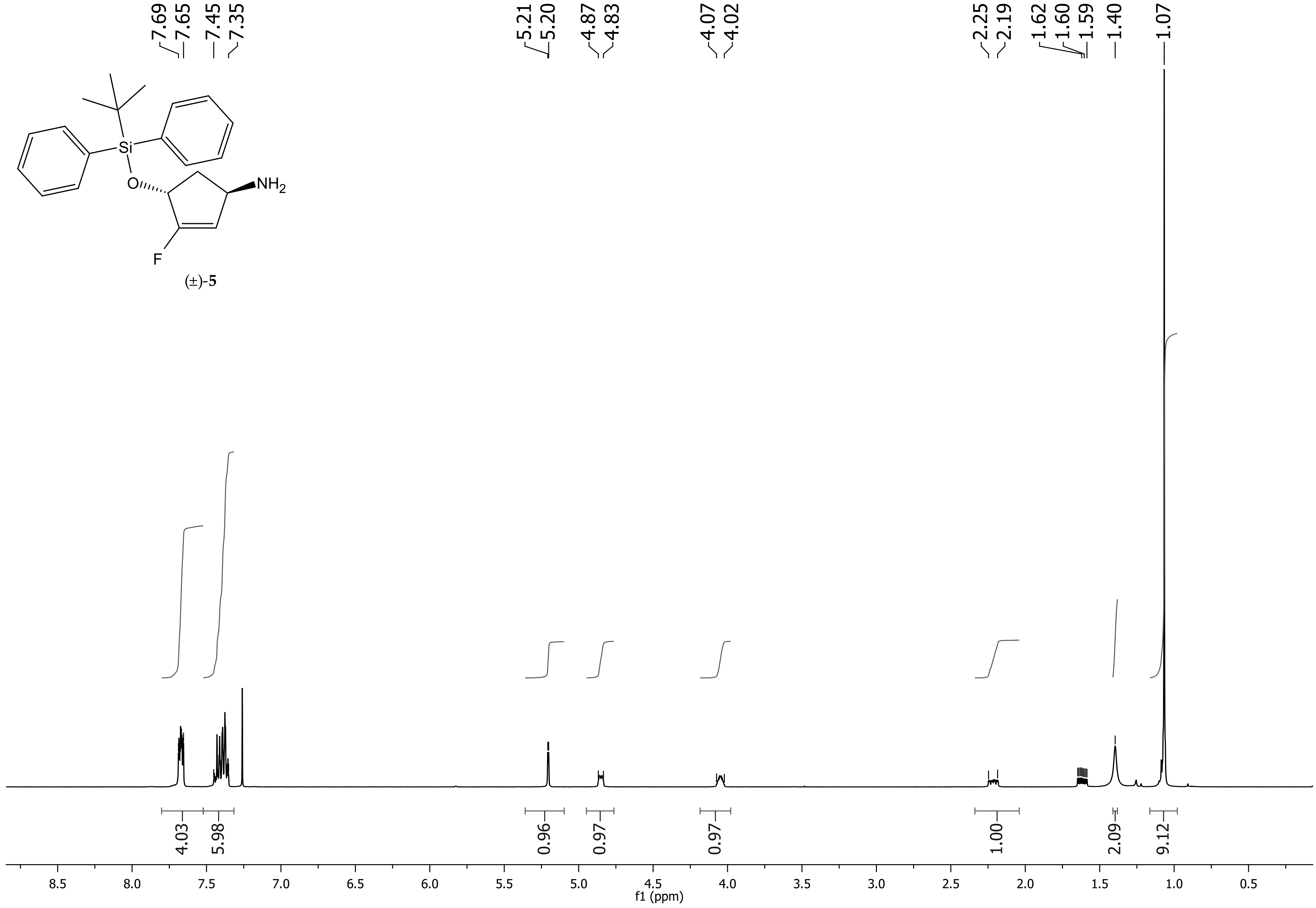
7.69
7.65
7.45
7.35
5.21
5.20
4.87
4.83
4.07
4.02
2.25
2.19
1.62
1.60
1.59
1.40
1.07
Si
O
NH2
F
(±)-5
4.03
5.98
0.96
0.97
0.97
1.00
2.09
9.12
8.5
8.0
7.5
7.0
6.5
6.0
5.5
5.0
4.5
4.0
3.5
3.0
2.5
2.0
1.5
1.0
0.5
f1 (ppm)

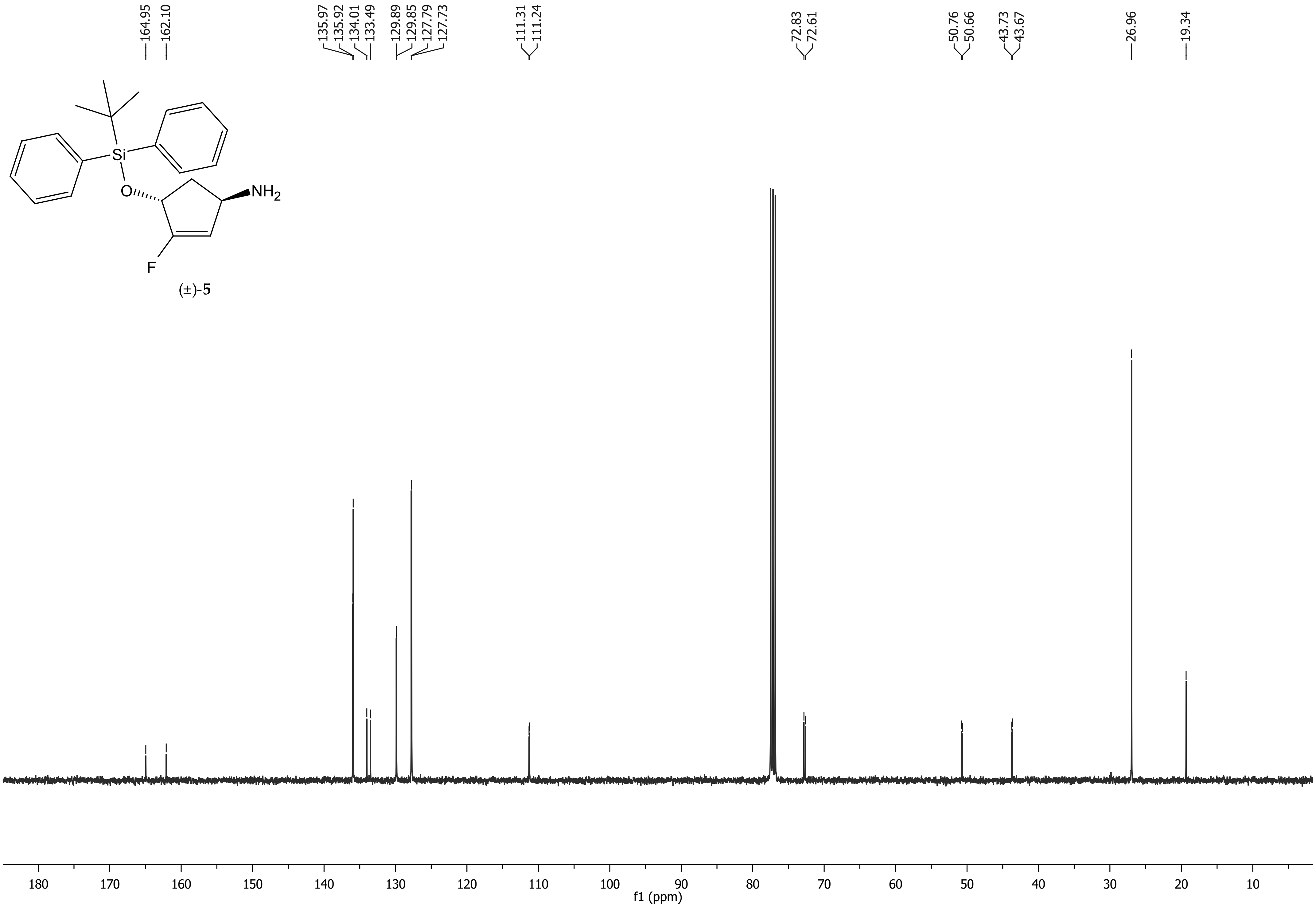
164.95
162.10
135.97
135.92
134.01
133.49
129.89
129.85
127.79
127.73
111.31
111.24
72.83
72.61
50.76
50.66
43.73
43.67
26.96
19.34
Si
O
NH2
F
(±)-5
180 170 160 150 140 130 120 110 100 90 80 70 60 50 40 30 20 10
f1 (ppm)

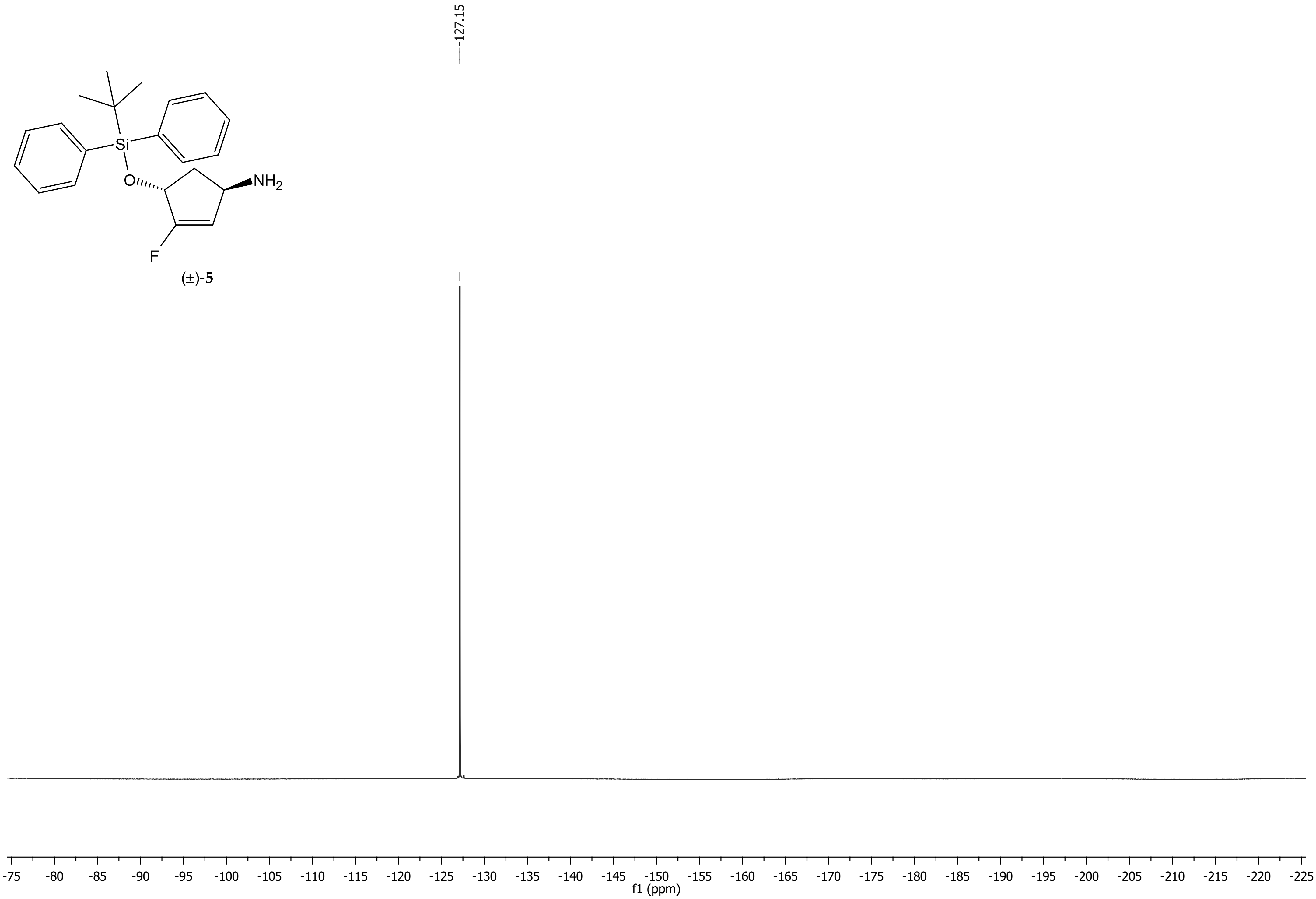
-127.15
Si
O
NH2
F
(±)-5
-75 -80 -85 -90 -95 -100 -105 -110 -115 -120 -125 -130 -135 -140 -145 -150 -155 -160 -165 -170 -175 -180 -185 -190 -195 -200 -205 -210 -215 -220 -225
f1 (ppm)

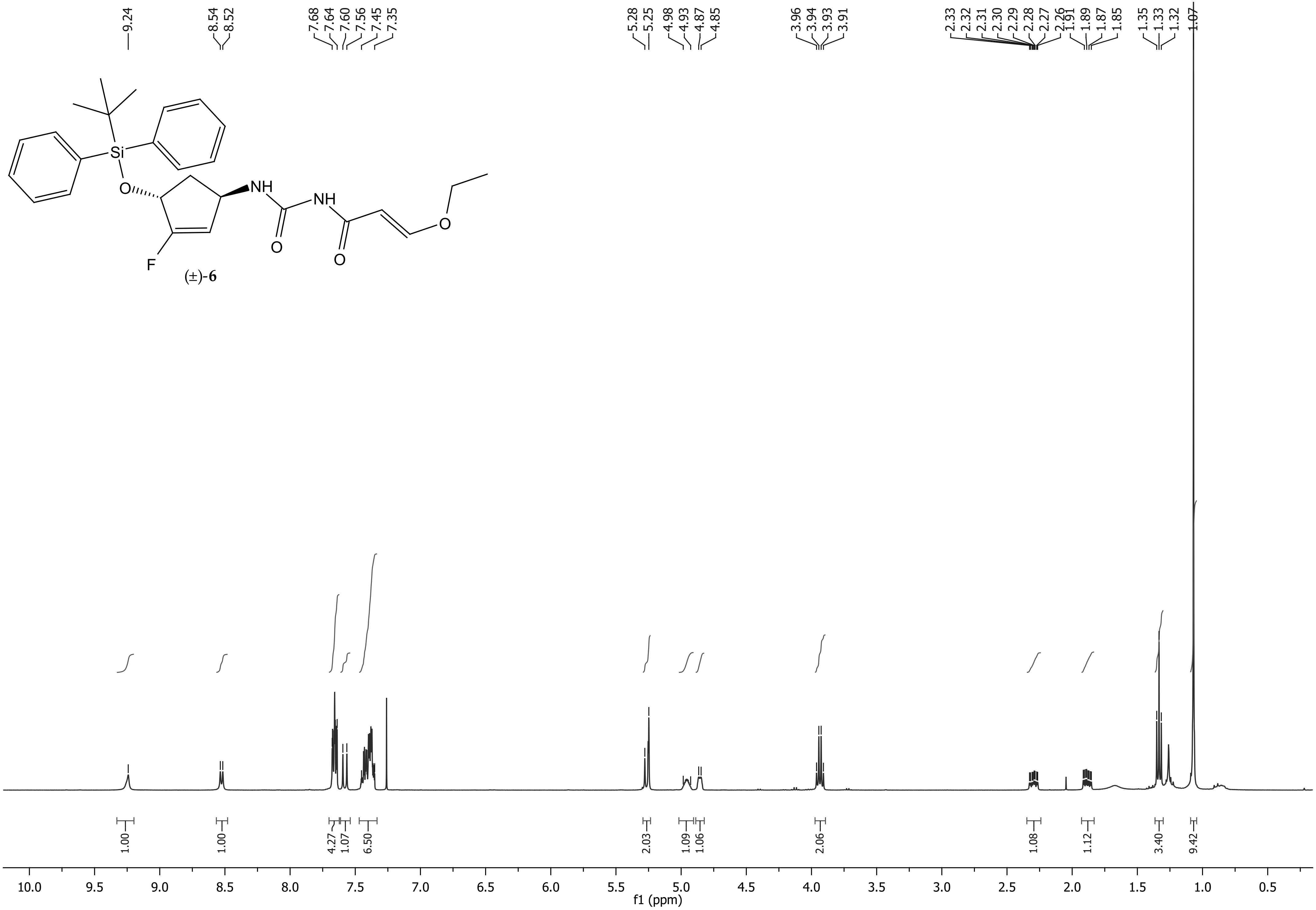
9.24
8.54
8.52
7.68
7.64
7.60
7.56
7.45
7.35
5.28
5.25
4.98
4.93
4.87
4.85
3.96
3.94
3.93
3.91
2.33
2.32
2.31
2.30
2.29
2.28
2.27
2.26
1.91
1.89
1.87
1.85
1.35
1.33
1.32
1.07
Si
O
NH
NH
O
O
O
F
(±)-6
1.00
1.00
4.27
1.07
6.50
2.03
1.09
1.06
2.06
1.08
1.12
3.40
9.42
10.0
9.5
9.0
8.5
8.0
7.5
7.0
6.5
6.0
5.5
5.0
4.5
4.0
3.5
3.0
2.5
2.0
1.5
1.0
0.5
f1 (ppm)

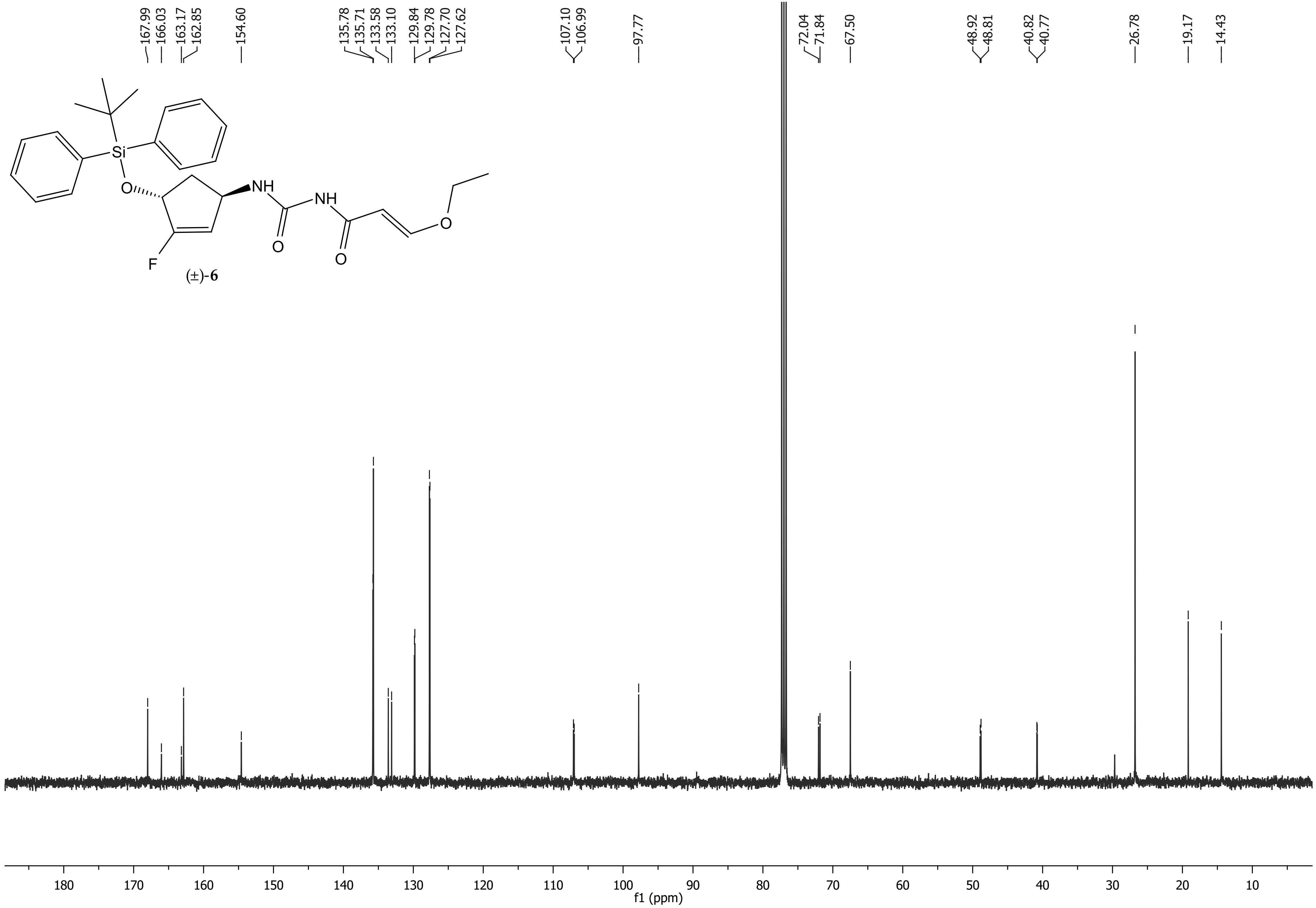
167.99
166.03
163.17
162.85
154.60
135.78
135.71
133.58
133.10
129.84
129.78
127.70
127.62
107.10
106.99
97.77
72.04
71.84
67.50
48.92
48.81
40.82
40.77
26.78
19.17
14.43
Si
O
NH
NH
O
O
O
F
(±)-6
180
170
160
150
140
130
120
110
100
90
80
70
60
50
40
30
20
10
f1 (ppm)

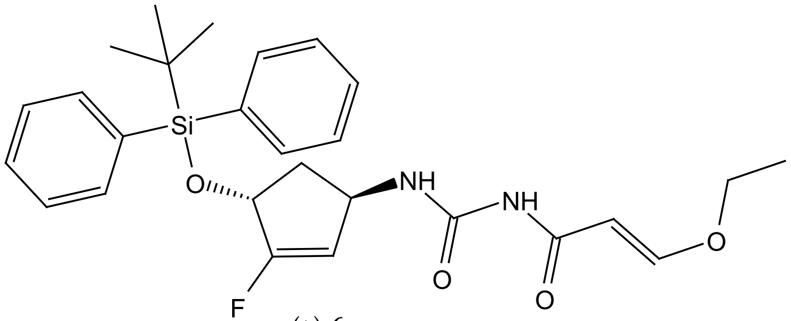

Si
O
NH
NH
O
O
O
F

(±)-**6**

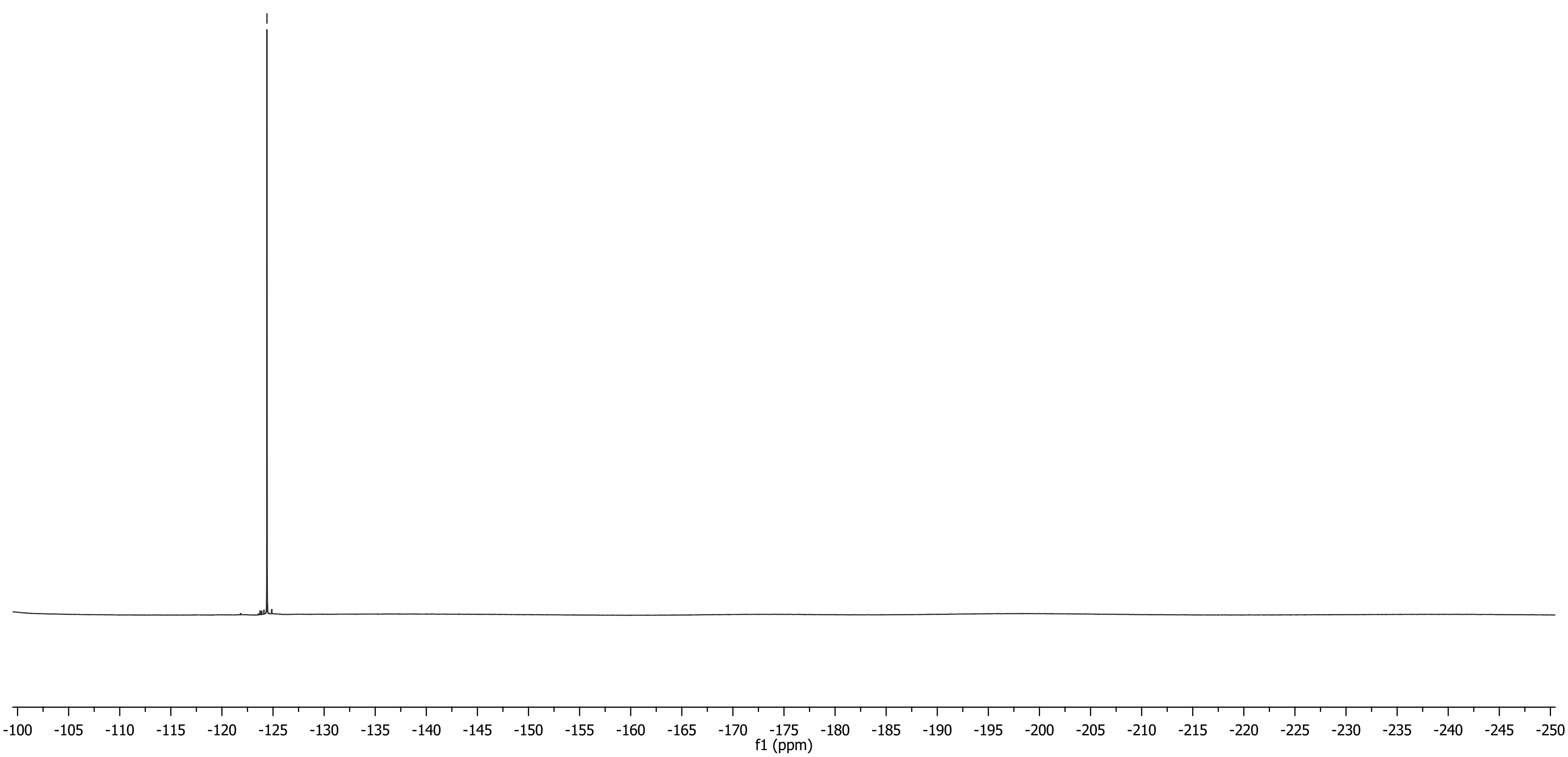

-124.41
-100 -105 -110 -115 -120 -125 -130 -135 -140 -145 -150 -155 -160 -165 -170 -175 -180 -185 -190 -195 -200 -205 -210 -215 -220 -225 -230 -235 -240 -245 -250
f1 (ppm)

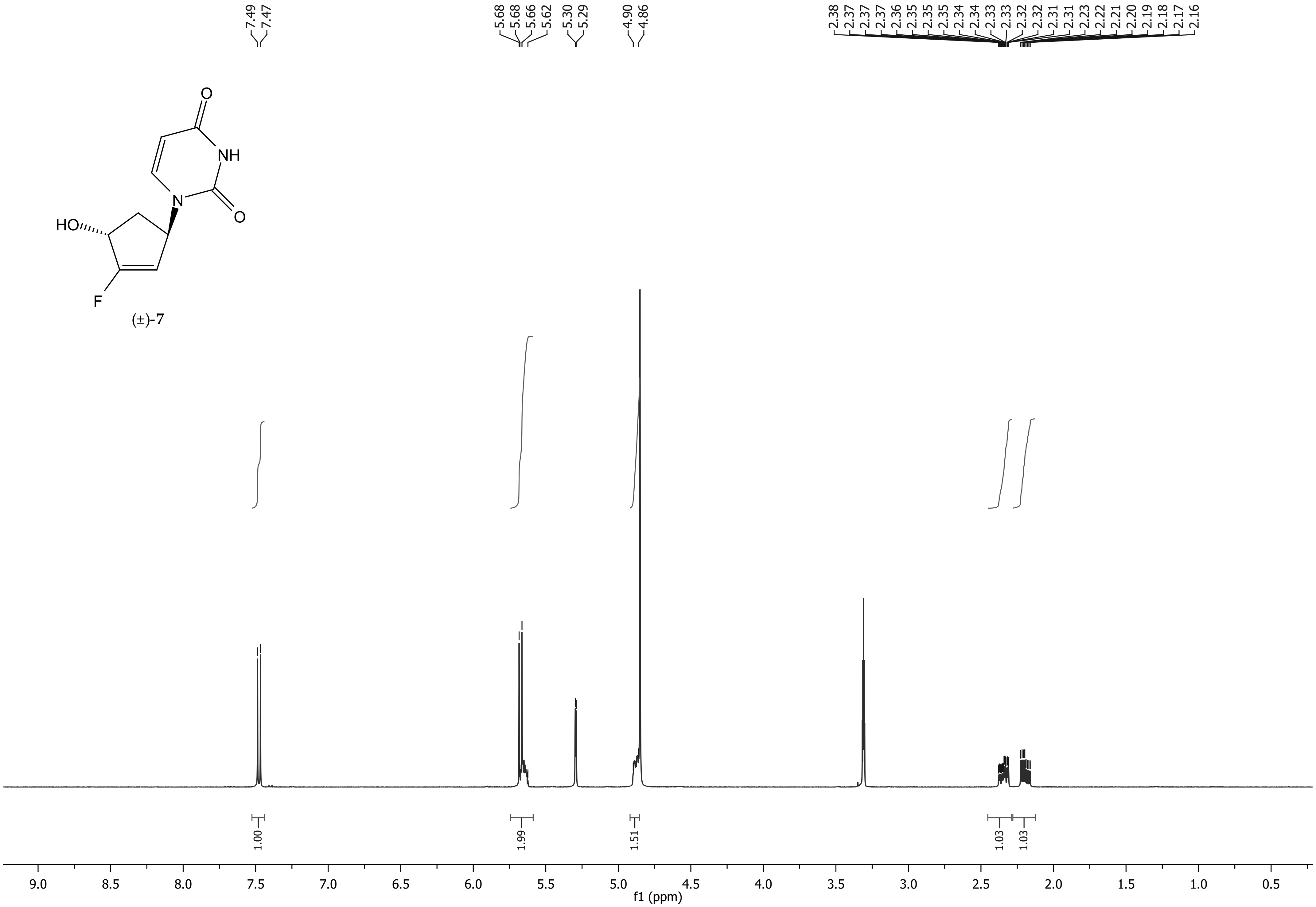
7.49
7.47
5.68
5.68
5.66
5.62
5.30
5.29
4.90
4.86
2.38
2.37
2.37
2.37
2.36
2.35
2.35
2.35
2.34
2.34
2.33
2.33
2.32
2.32
2.31
2.31
2.23
2.22
2.21
2.20
2.19
2.18
2.17
2.16
O
NH
N
O
HO
F
(±)-7
1.00
1.99
1.51
1.03
1.03
9.0
8.5
8.0
7.5
7.0
6.5
6.0
5.5
5.0
4.5
4.0
3.5
3.0
2.5
2.0
1.5
1.0
0.5
f1 (ppm)

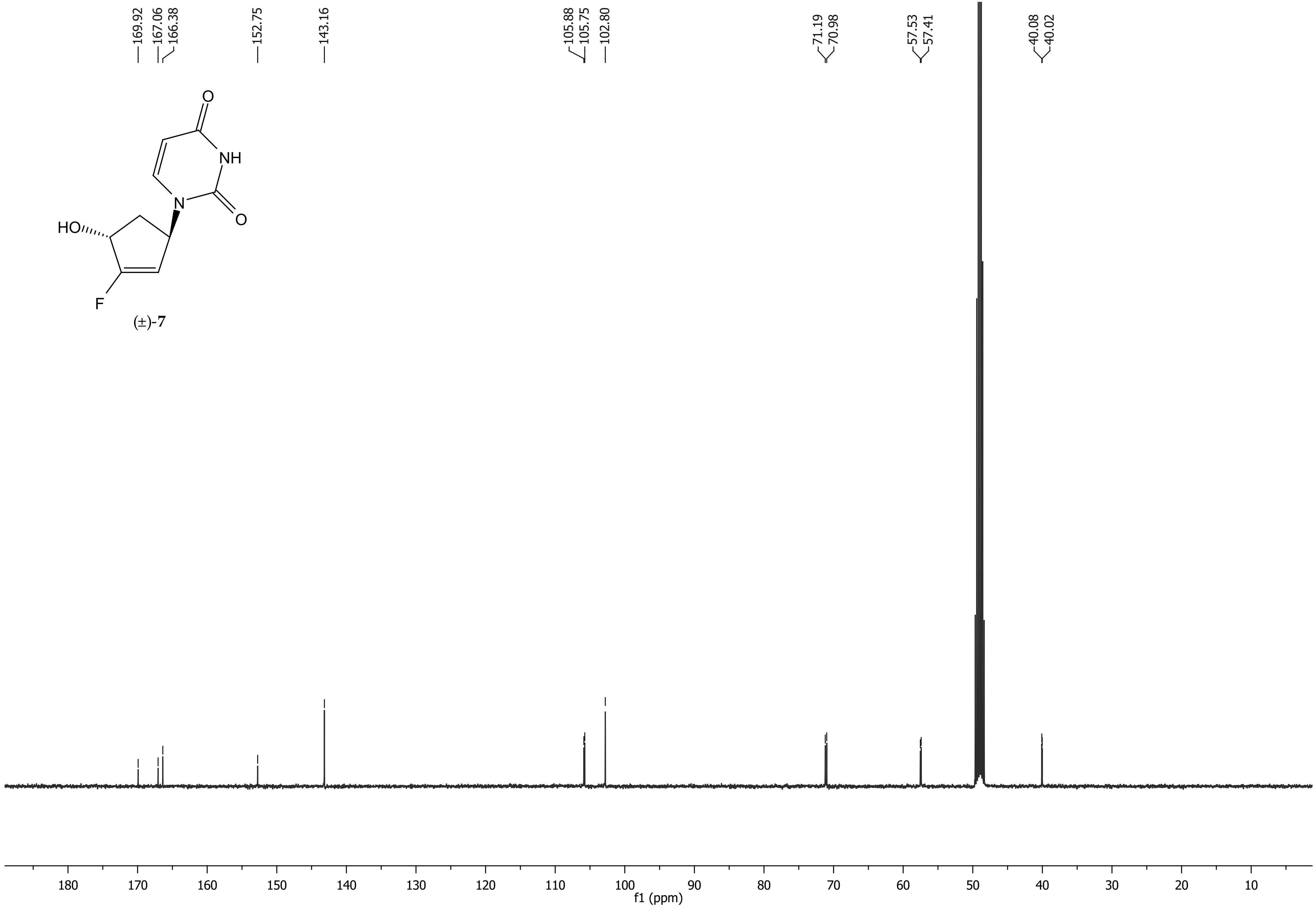
169.92
167.06
166.38
152.75
143.16
105.88
105.75
102.80
71.19
70.98
57.53
57.41
40.08
40.02
O
NH
N
O
HO
F
(±)-7
180 170 160 150 140 130 120 110 100 90 80 70 60 50 40 30 20 10
f1 (ppm)

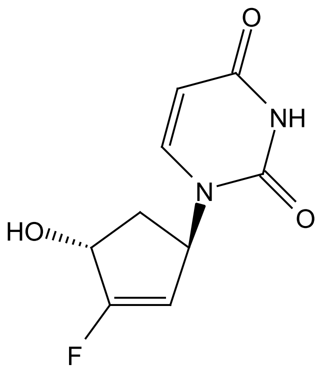
O
NH
N
O
HO
F

(±)-7

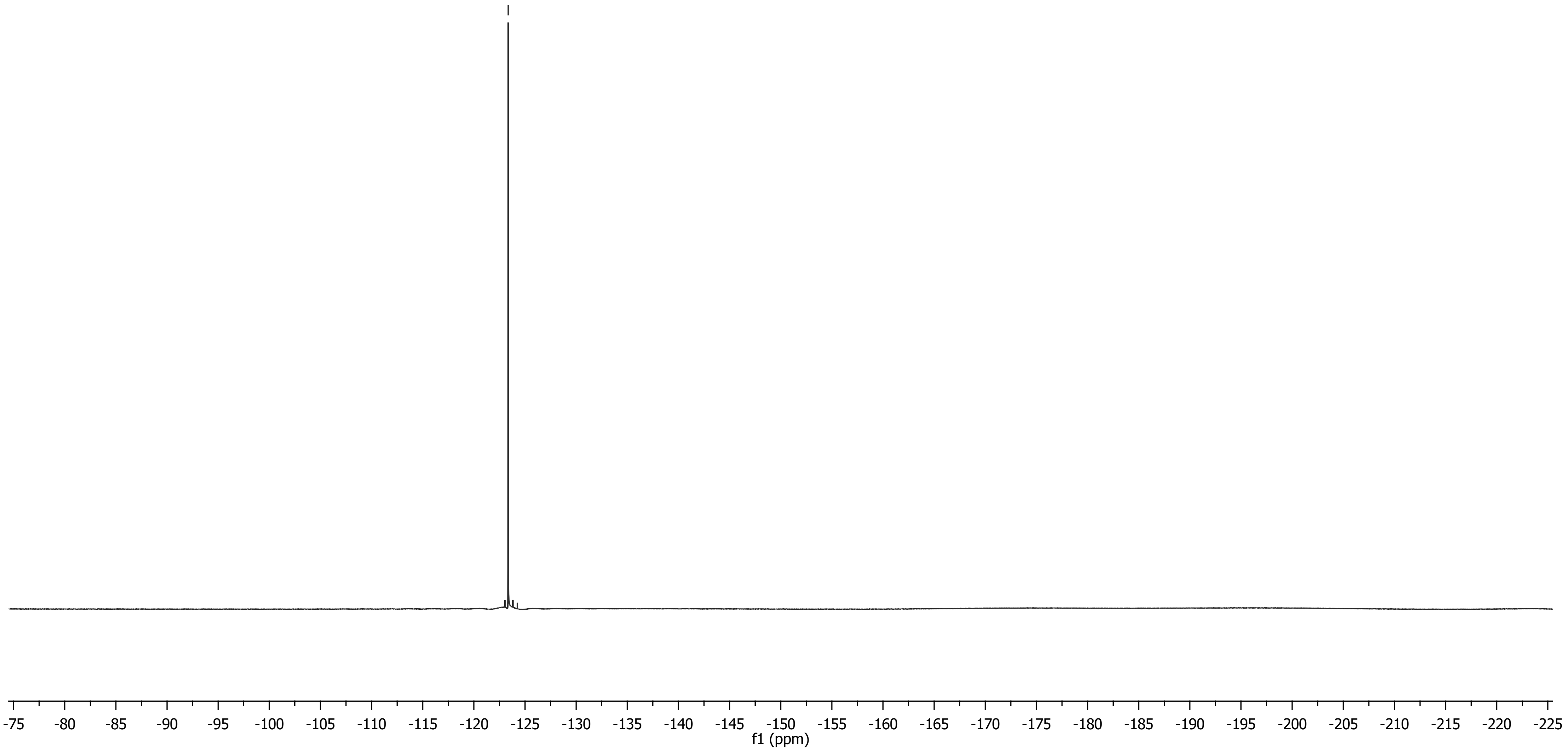
-123.35
-75 -80 -85 -90 -95 -100 -105 -110 -115 -120 -125 -130 -135 -140 -145 -150 -155 -160 -165 -170 -175 -180 -185 -190 -195 -200 -205 -210 -215 -220 -225
f1 (ppm)

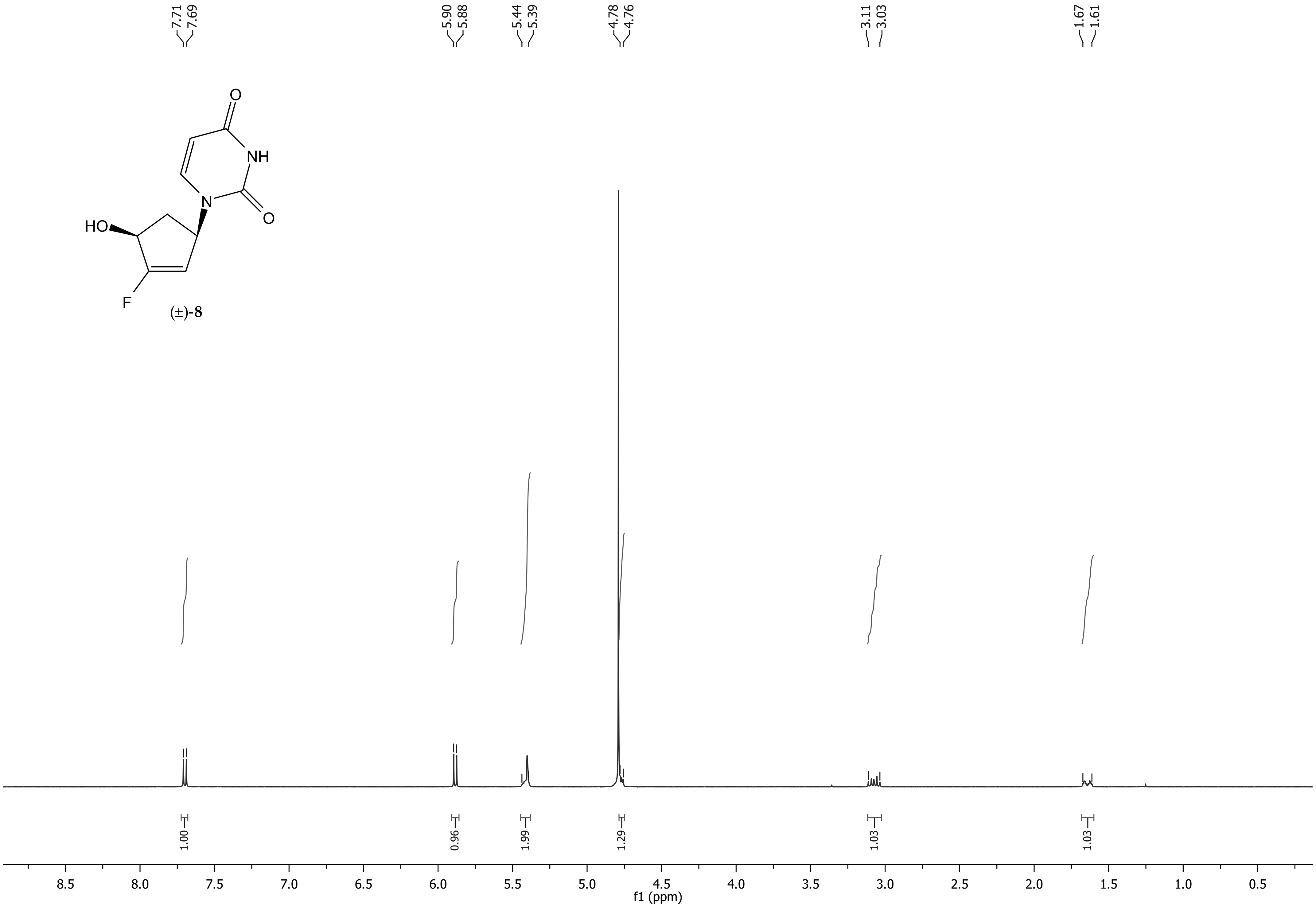

7.71
7.69
5.90
5.88
5.44
5.39
4.78
4.76
3.11
3.03
1.67
1.61
O
NH
N
O
HO
F
(±)-8
1.00
0.96
1.99
1.29
1.03
1.03
8.5
8.0
7.5
7.0
6.5
6.0
5.5
5.0
4.5
4.0
3.5
3.0
2.5
2.0
1.5
1.0
0.5
f1 (ppm)

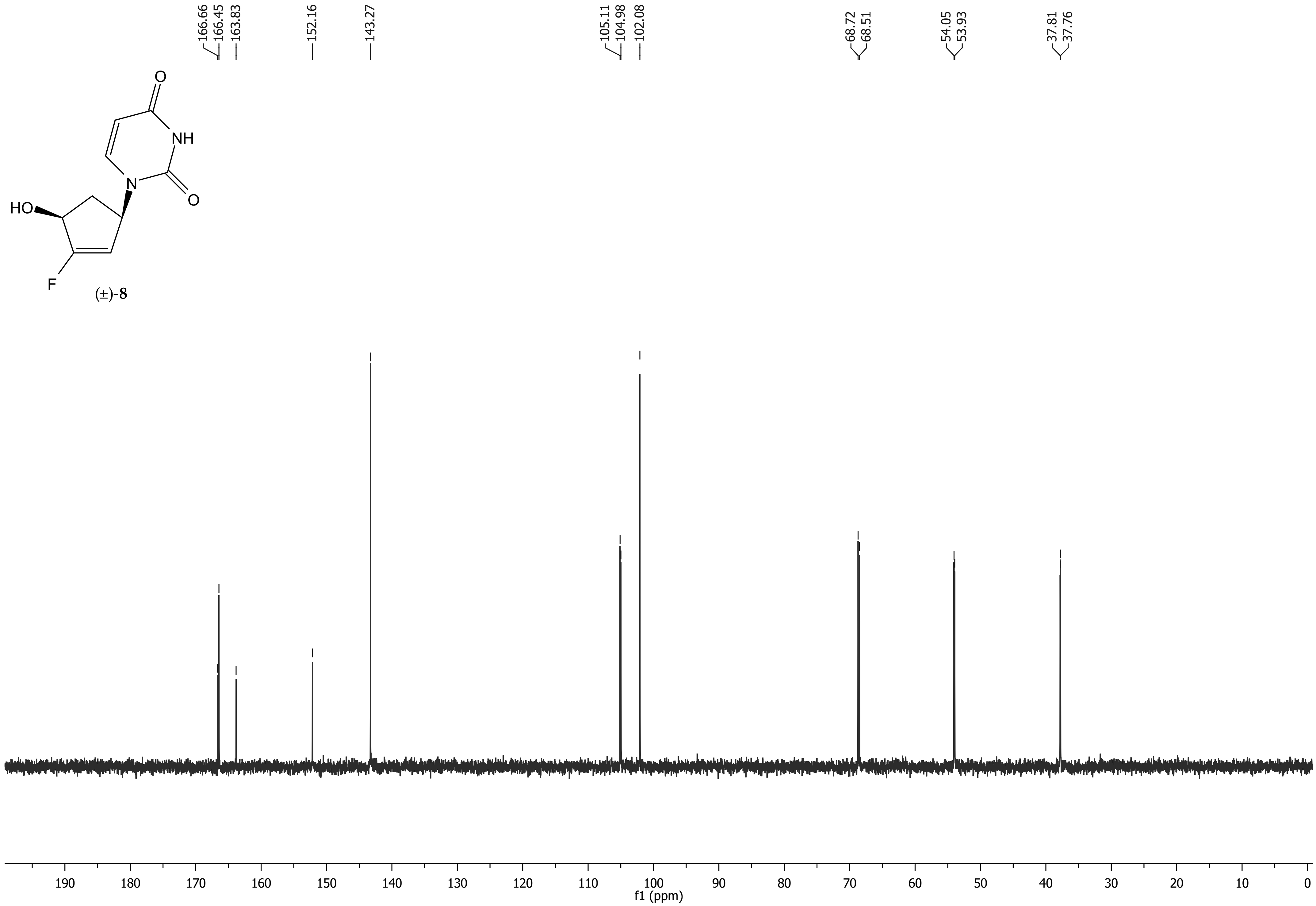
166.66
166.45
163.83
152.16
143.27
105.11
104.98
102.08
68.72
68.51
54.05
53.93
37.81
37.76
O
NH
N
O
HO
F
(±)-8
190 180 170 160 150 140 130 120 110 100 90 80 70 60 50 40 30 20 10 0
f1 (ppm)

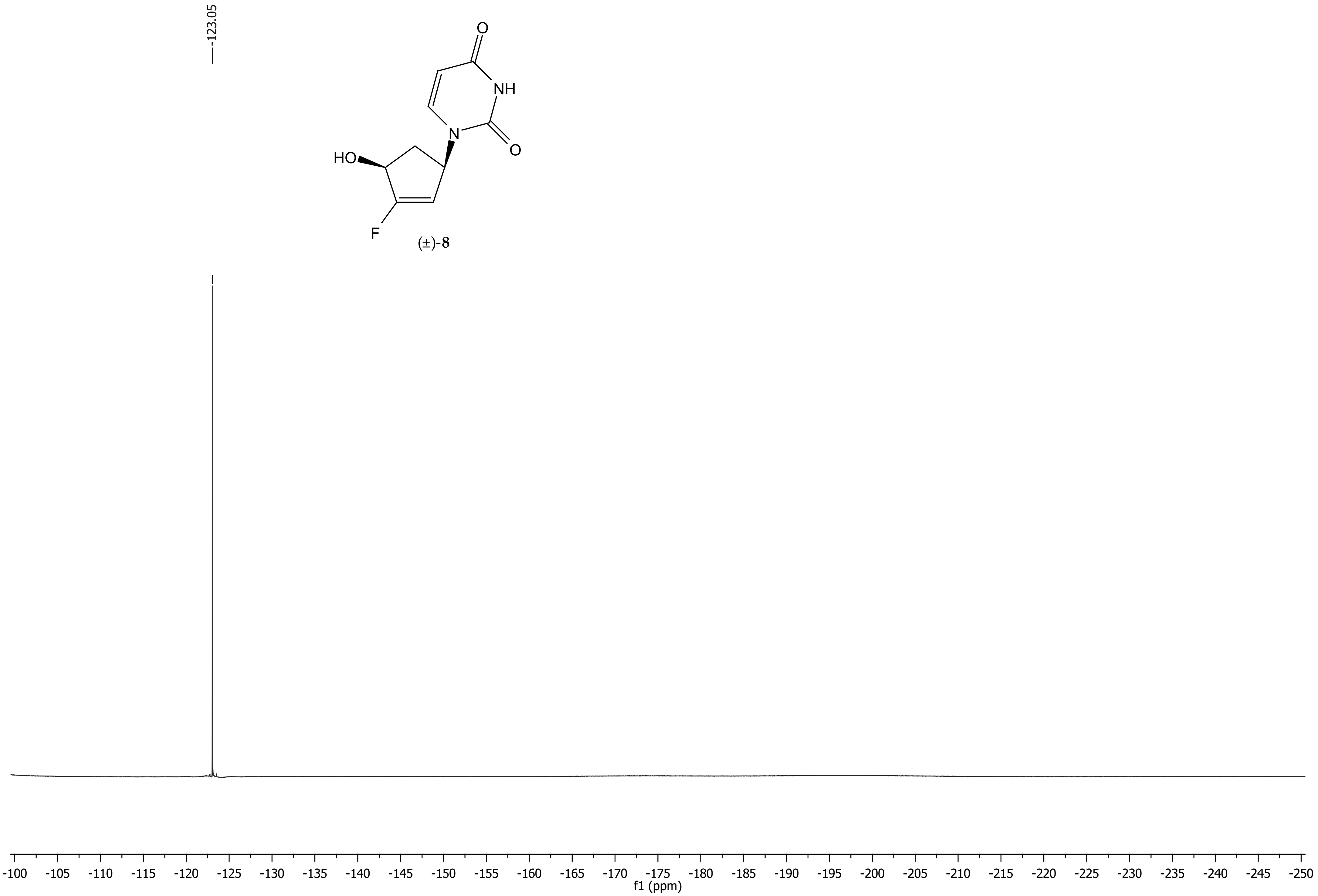
-123.05
HO
F
N
NH
O
O
(±)-8
-100 -105 -110 -115 -120 -125 -130 -135 -140 -145 -150 -155 -160 -165 -170 -175 -180 -185 -190 -195 -200 -205 -210 -215 -220 -225 -230 -235 -240 -245 -250
f1 (ppm)

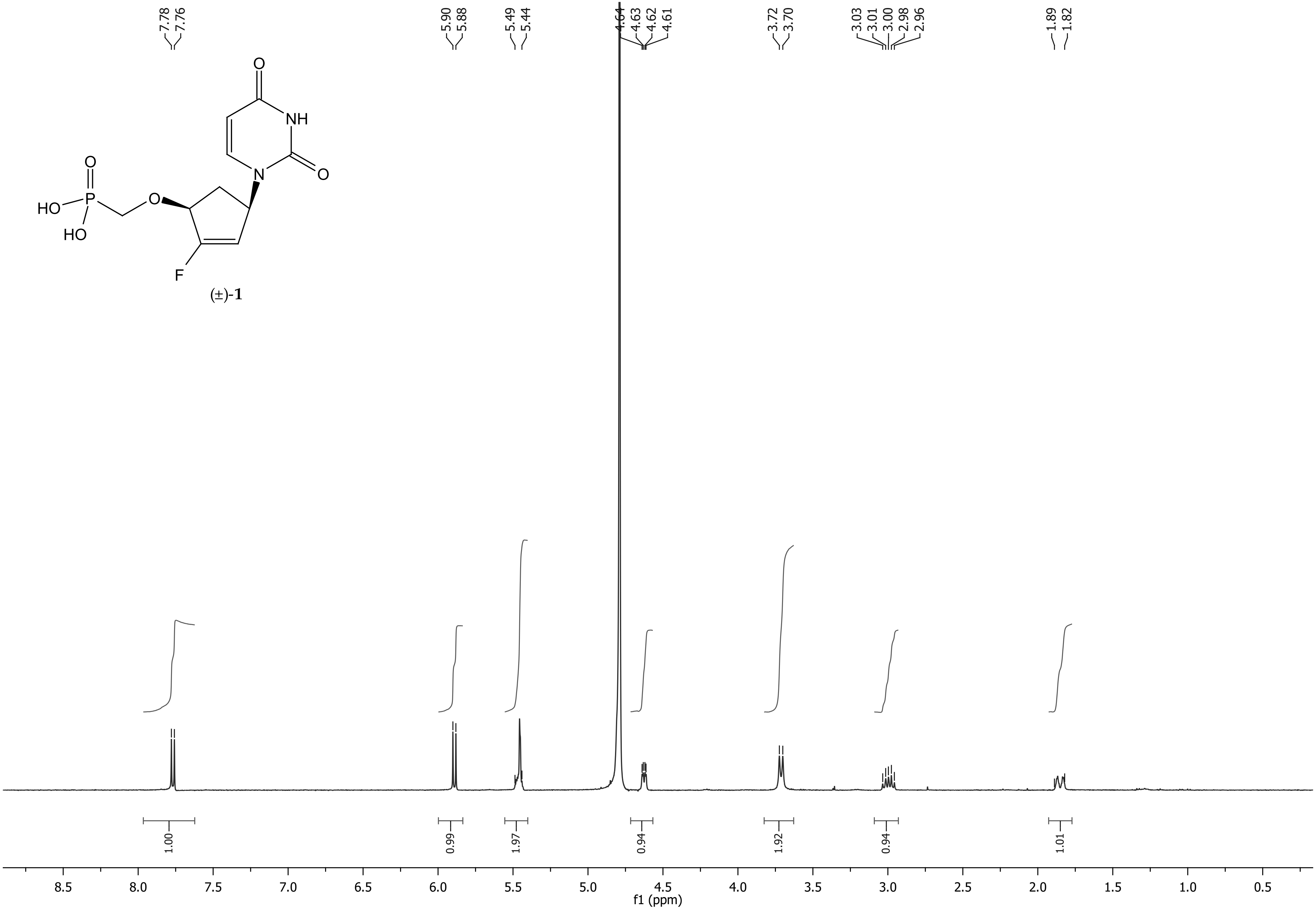

7.78
7.76
5.90
5.88
5.49
5.44
4.64
4.63
4.62
4.61
3.72
3.70
3.03
3.01
3.00
2.98
2.96
1.89
1.82
O
NH
O
N
O
HO
P
O
HO
F
(±)-1
1.00
0.99
1.97
0.94
1.92
0.94
1.01
8.5
8.0
7.5
7.0
6.5
6.0
5.5
5.0
4.5
4.0
3.5
3.0
2.5
2.0
1.5
1.0
0.5
f1 (ppm)

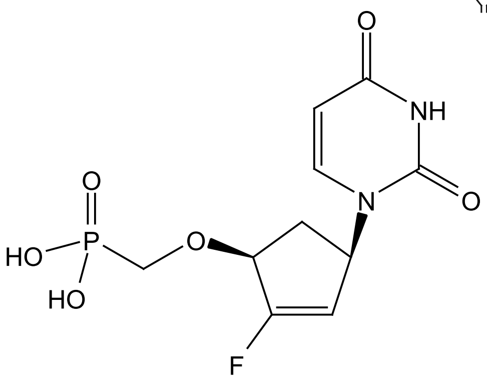
O
NH
O
N
O
HO
P
O
HO
F

(±)-**1**

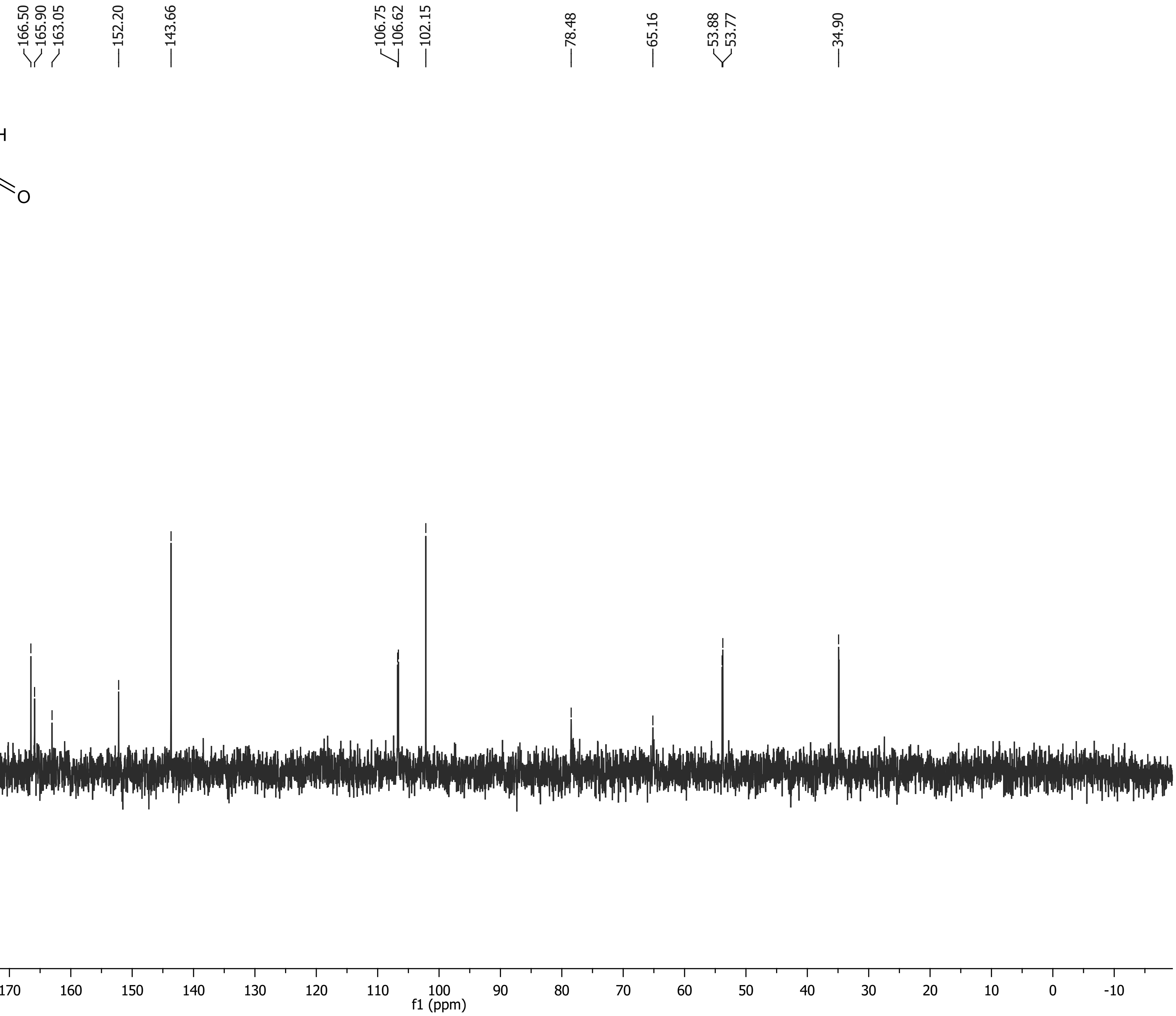
166.50
165.90
163.05
152.20
143.66
106.75
106.62
102.15
78.48
65.16
53.88
53.77
34.90
210
200
190
180
170
160
150
140
130
120
110
100
90
80
70
60
50
40
30
20
10
0
-10
f1 (ppm)

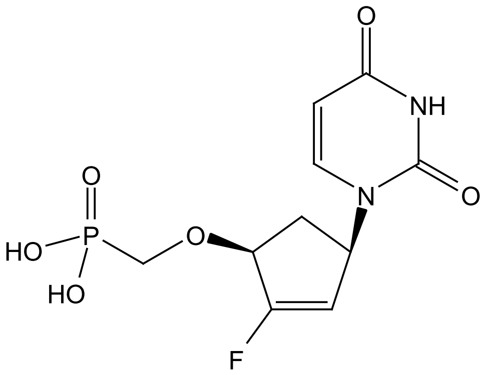
O
NH
O
N
O
HO
P
O
HO
F

(±)-**1**

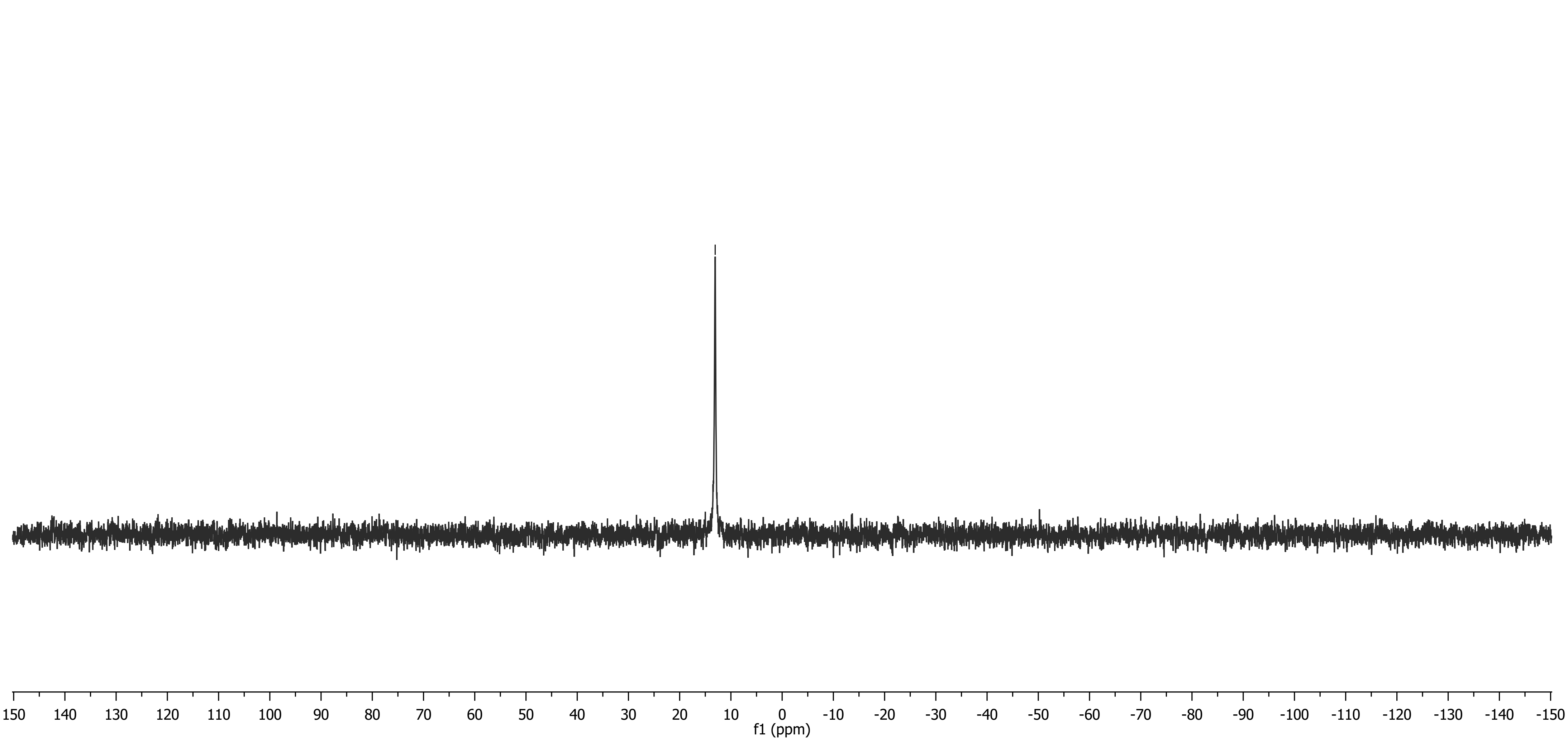
13.07
150 140 130 120 110 100 90 80 70 60 50 40 30 20 10 0 -10 -20 -30 -40 -50 -60 -70 -80 -90 -100 -110 -120 -130 -140 -150
f1 (ppm)

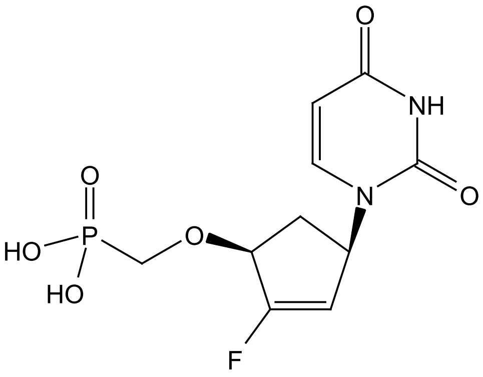

(±)-**1**

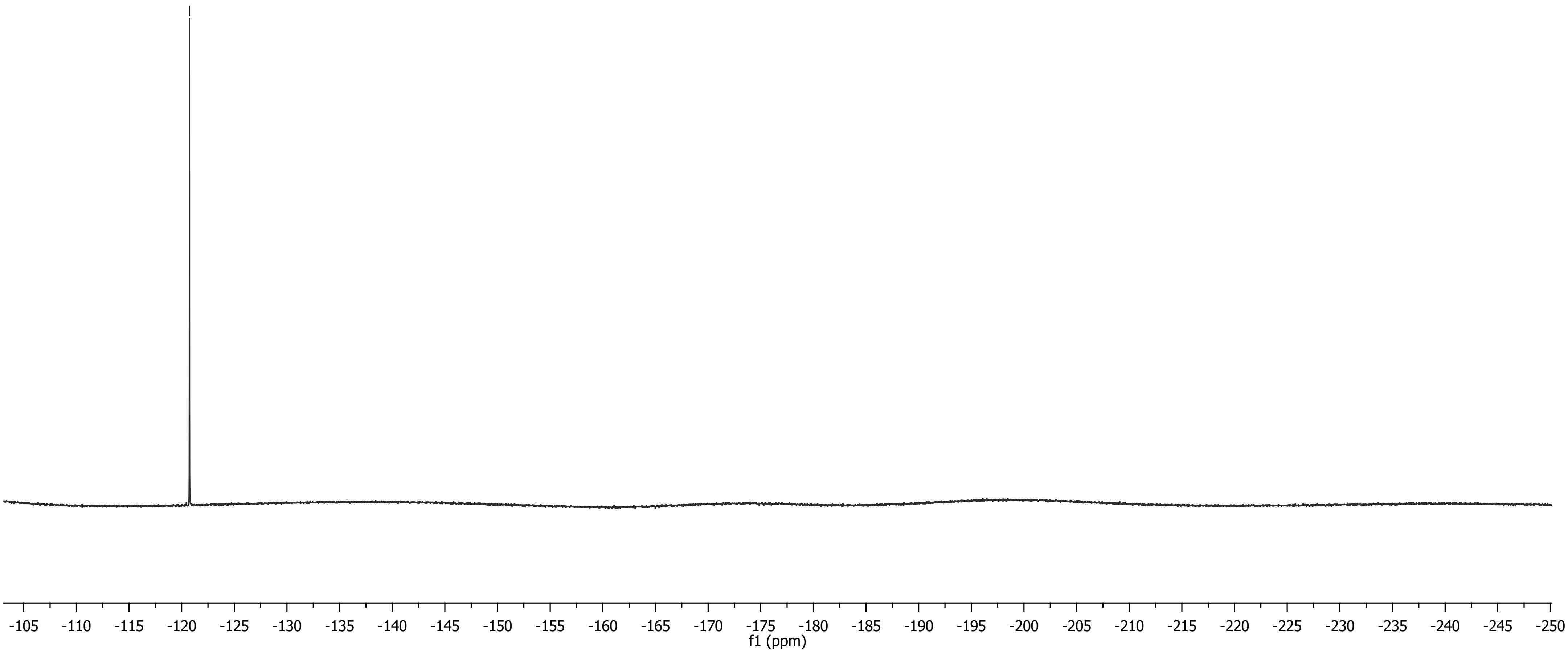
-120.74
-105 -110 -115 -120 -125 -130 -135 -140 -145 -150 -155 -160 -165 -170 -175 -180 -185 -190 -195 -200 -205 -210 -215 -220 -225 -230 -235 -240 -245 -250
f1 (ppm)

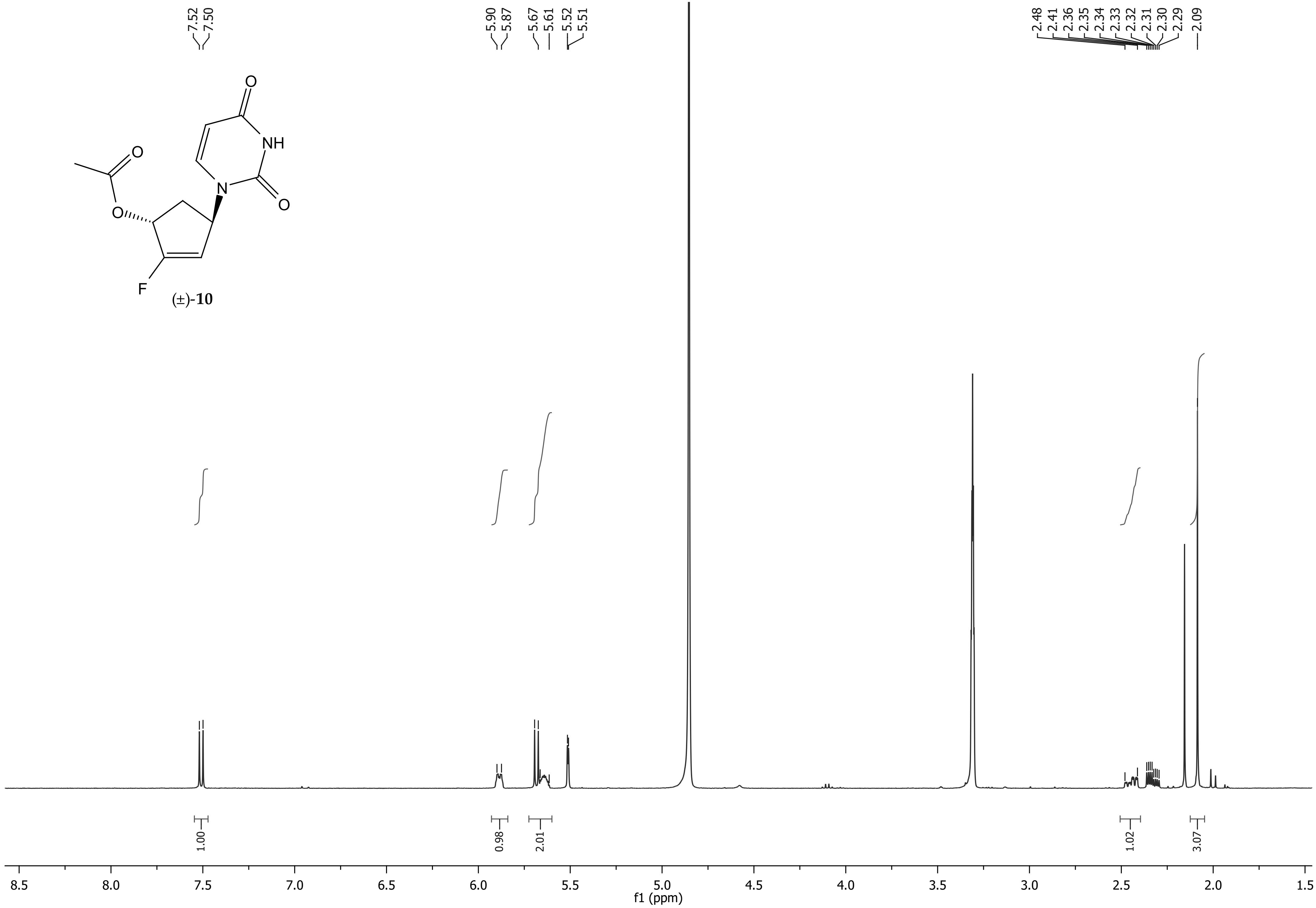
7.52
7.50
5.90
5.87
5.67
5.61
5.52
5.51
2.48
2.41
2.36
2.35
2.34
2.33
2.32
2.31
2.30
2.29
2.09
O
NH
O
N
O
O
O
F
(±)-10
1.00
0.98
2.01
1.02
3.07
8.5
8.0
7.5
7.0
6.5
6.0
5.5
5.0
4.5
4.0
3.5
3.0
2.5
2.0
1.5
f1 (ppm)

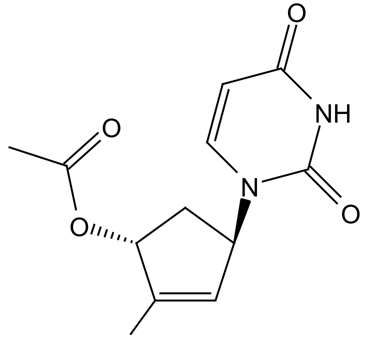
O
NH
N
O
O
O
F

(±)-**10**

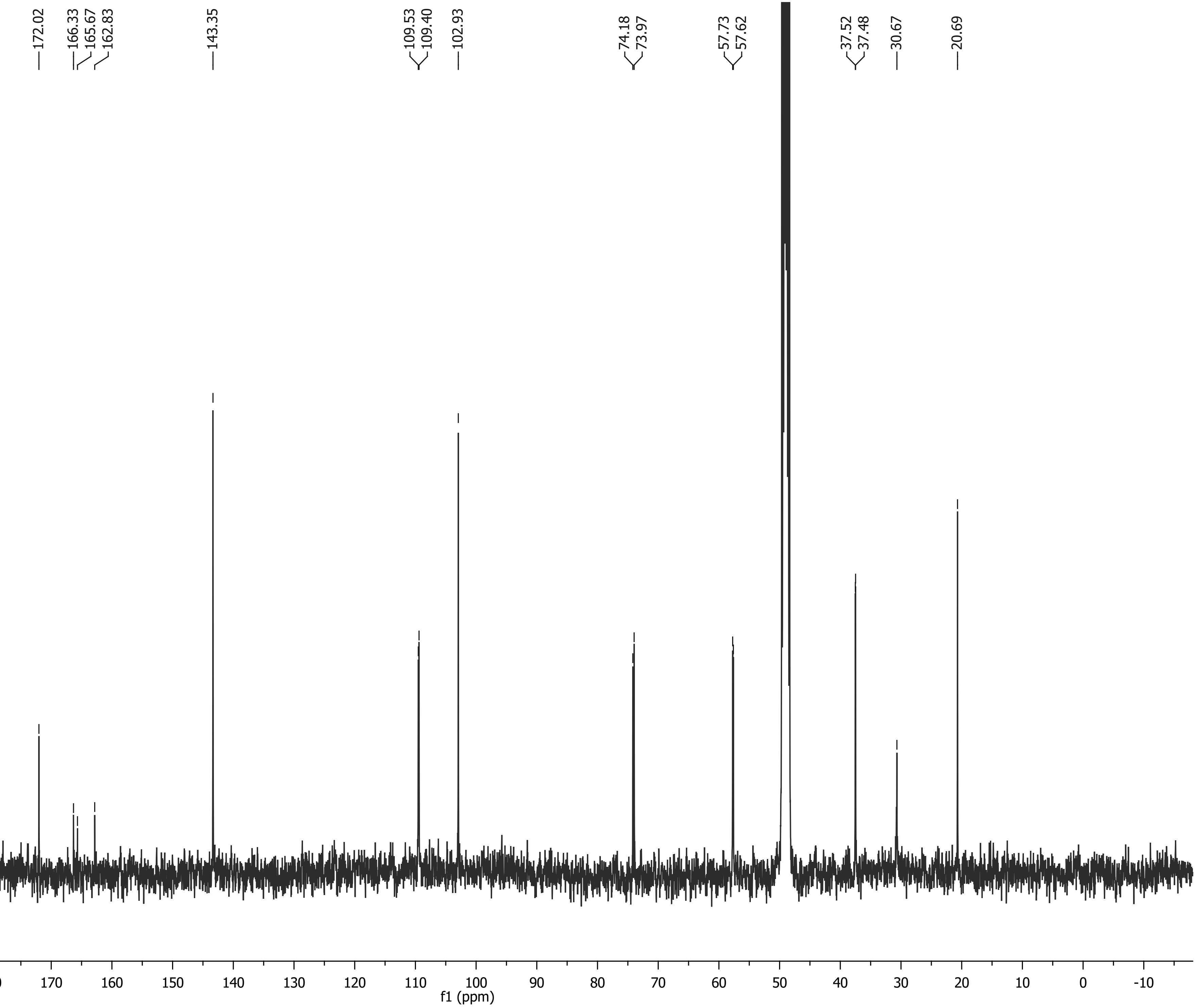
172.02
166.33
165.67
162.83
143.35
109.53
109.40
102.93
74.18
73.97
57.73
57.62
37.52
37.48
30.67
20.69
220 210 200 190 180 170 160 150 140 130 120 110 100 90 80 70 60 50 40 30 20 10 0 -10
f1 (ppm)

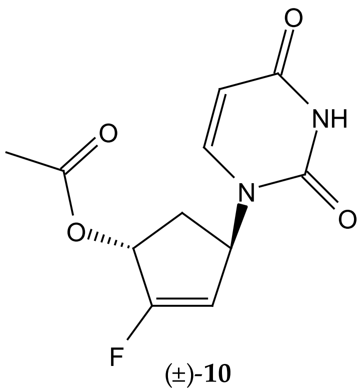

(±)-**10**

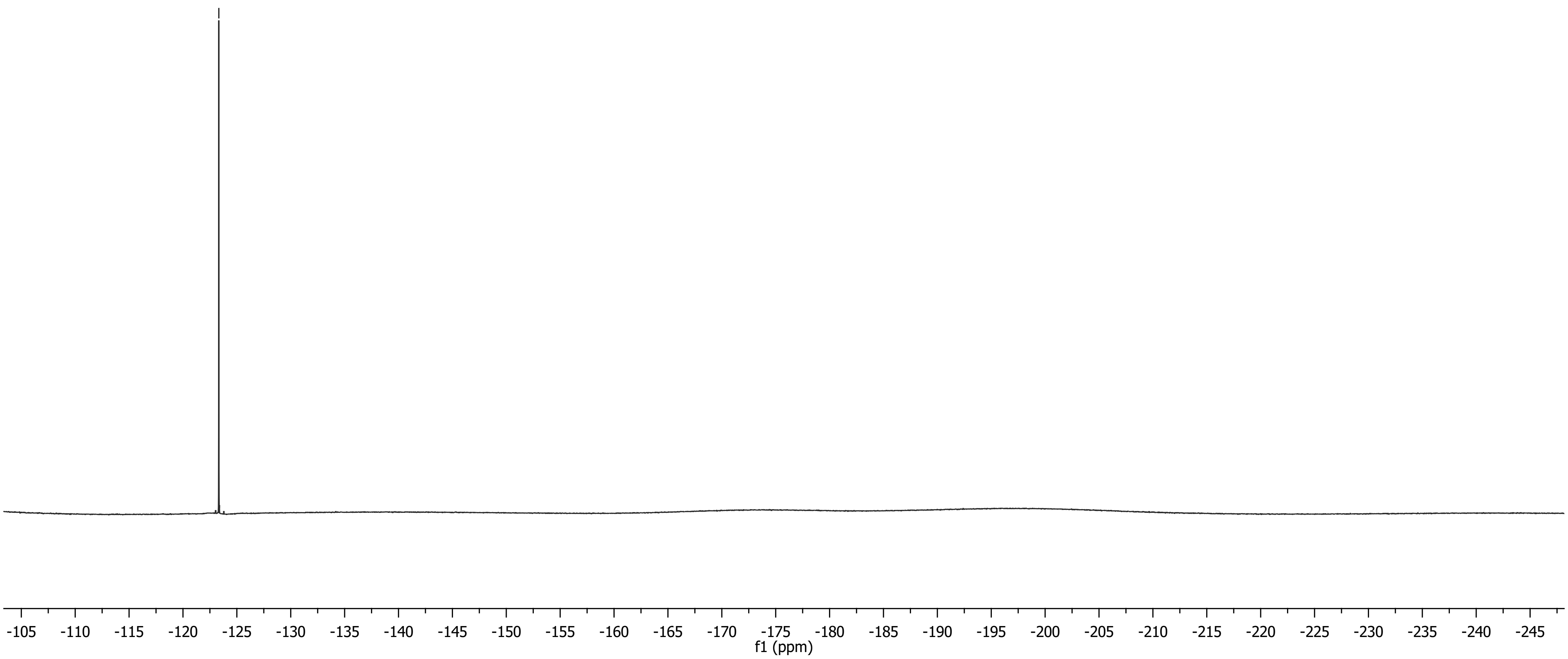
-123.33
-105 -110 -115 -120 -125 -130 -135 -140 -145 -150 -155 -160 -165 -170 -175 -180 -185 -190 -195 -200 -205 -210 -215 -220 -225 -230 -235 -240 -245
f1 (ppm)

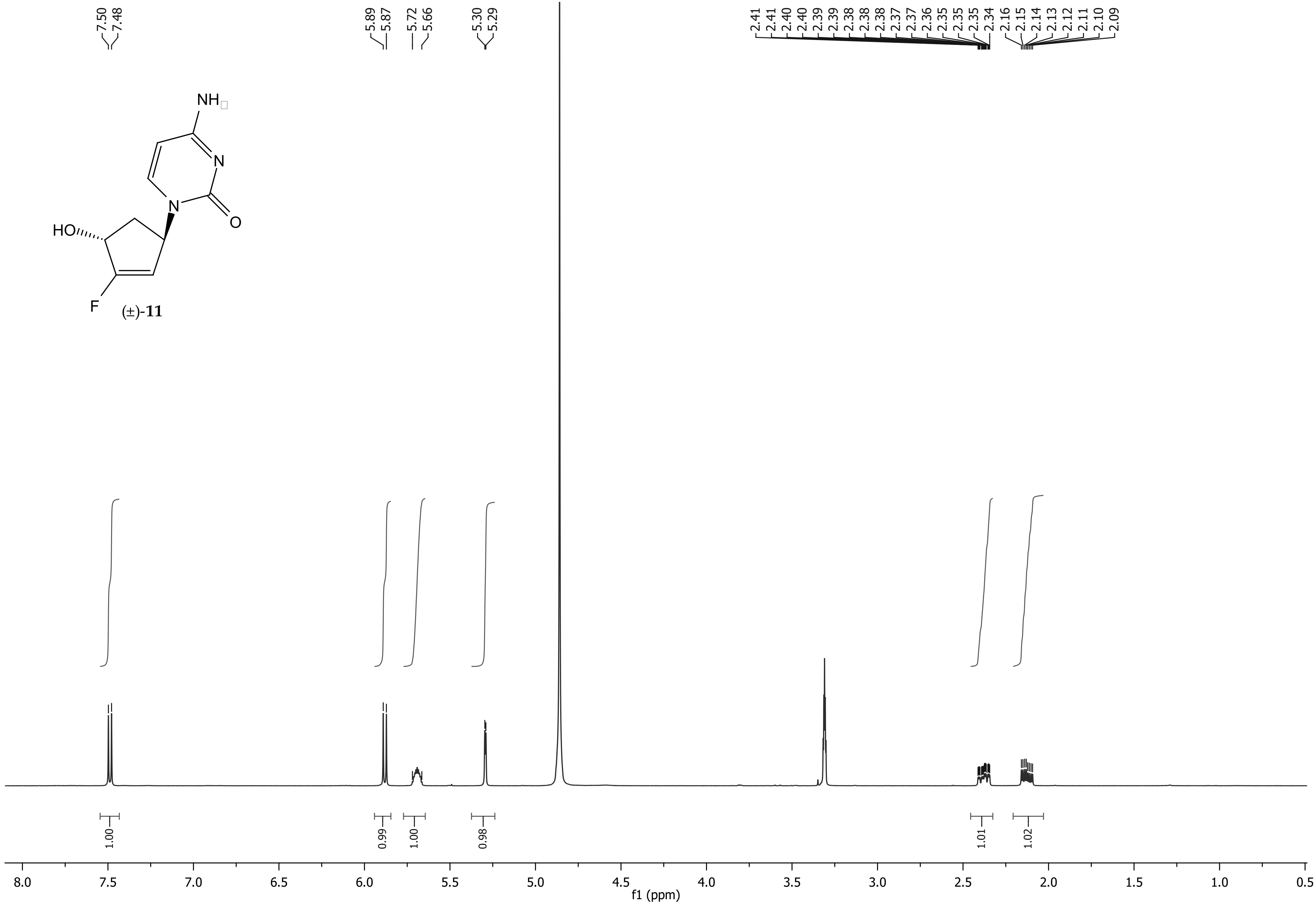
7.50
7.48
5.89
5.87
5.72
5.66
5.30
5.29
2.41
2.41
2.40
2.40
2.39
2.39
2.38
2.38
2.38
2.37
2.37
2.36
2.35
2.35
2.35
2.34
2.16
2.15
2.14
2.13
2.12
2.11
2.10
2.09
NH
N
N
O
HO
F
(±)-11
1.00
0.99
1.00
0.98
1.01
1.02
8.0
7.5
7.0
6.5
6.0
5.5
5.0
4.5
4.0
3.5
3.0
2.5
2.0
1.5
1.0
0.5
f1 (ppm)

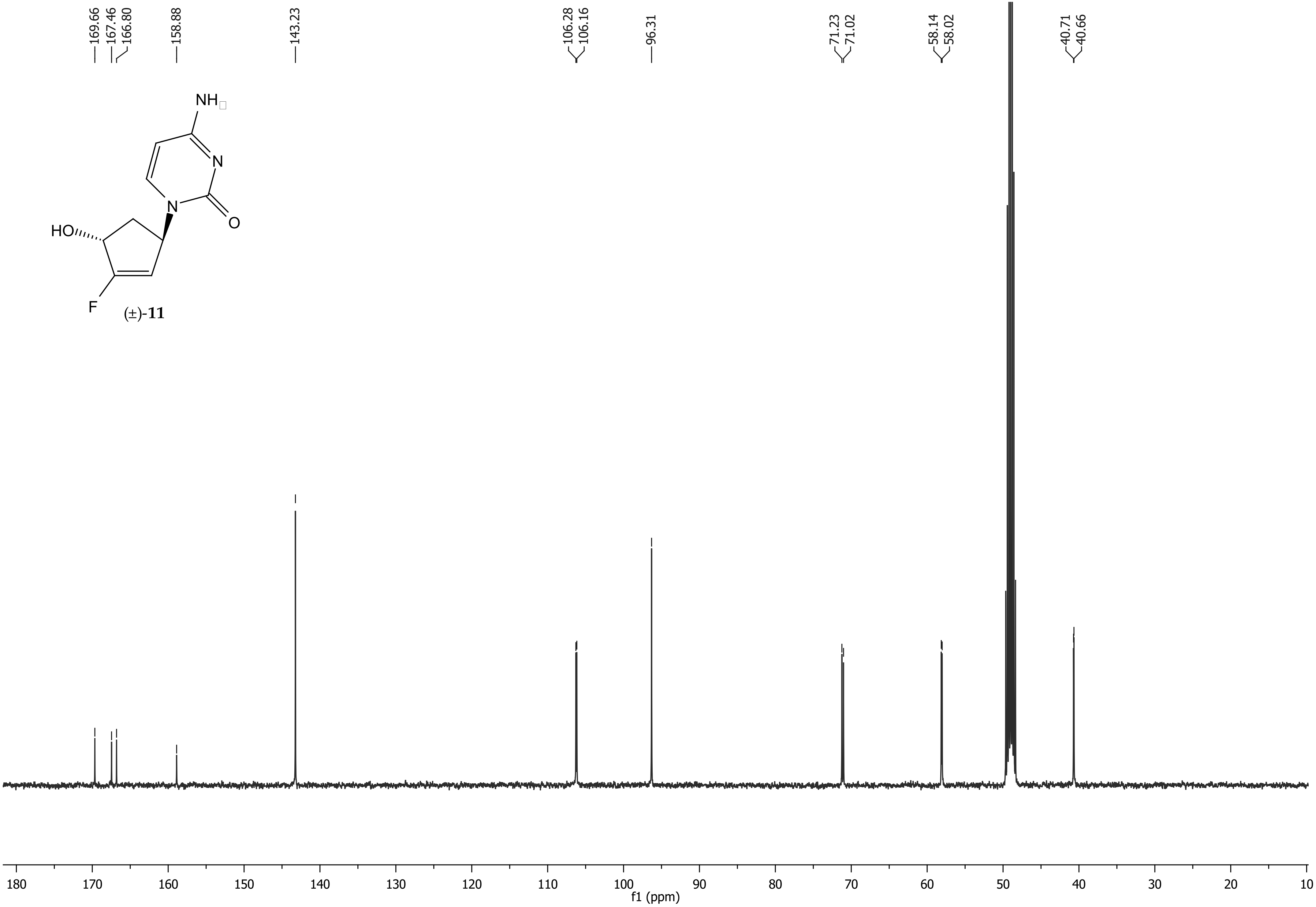
169.66
167.46
166.80
158.88
143.23
106.28
106.16
96.31
71.23
71.02
58.14
58.02
40.71
40.66
NH₂
N
O
HO
F
(±)-11
180 170 160 150 140 130 120 110 100 90 80 70 60 50 40 30 20 10
f1 (ppm)

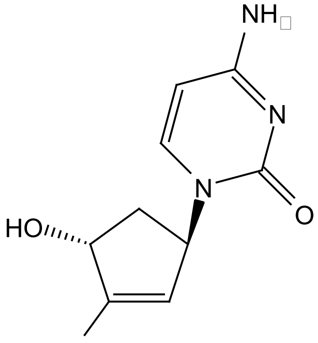

(±)-**11**

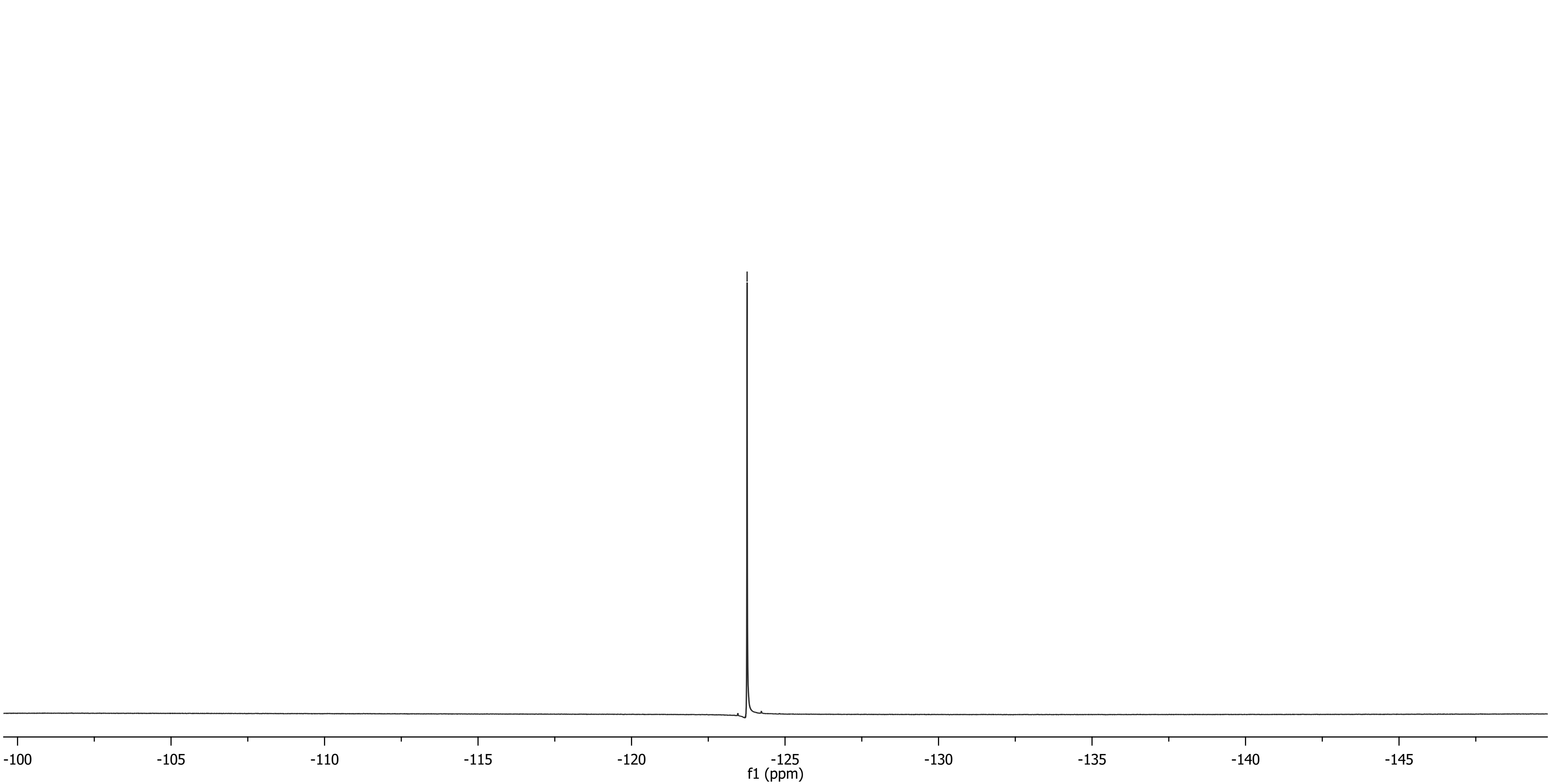

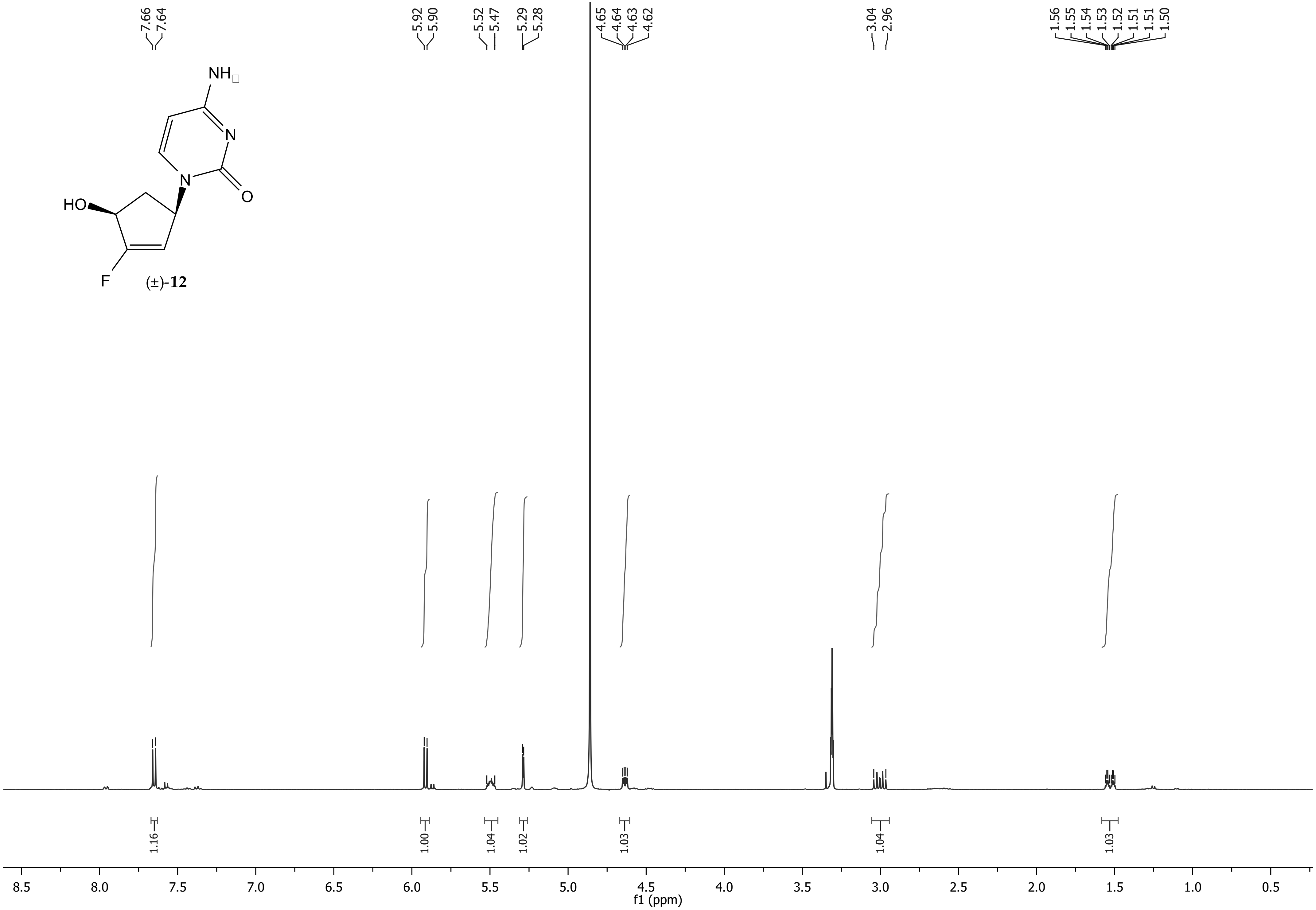
7.66
7.64
5.92
5.90
5.52
5.47
5.29
5.28
4.65
4.64
4.63
4.62
3.04
2.96
1.56
1.55
1.54
1.53
1.52
1.51
1.51
1.50
NH
N
O
N
HO
F
(±)-12
1.16
1.00
1.04
1.02
1.03
1.04
1.03
8.5
8.0
7.5
7.0
6.5
6.0
5.5
5.0
4.5
4.0
3.5
3.0
2.5
2.0
1.5
1.0
0.5
f1 (ppm)

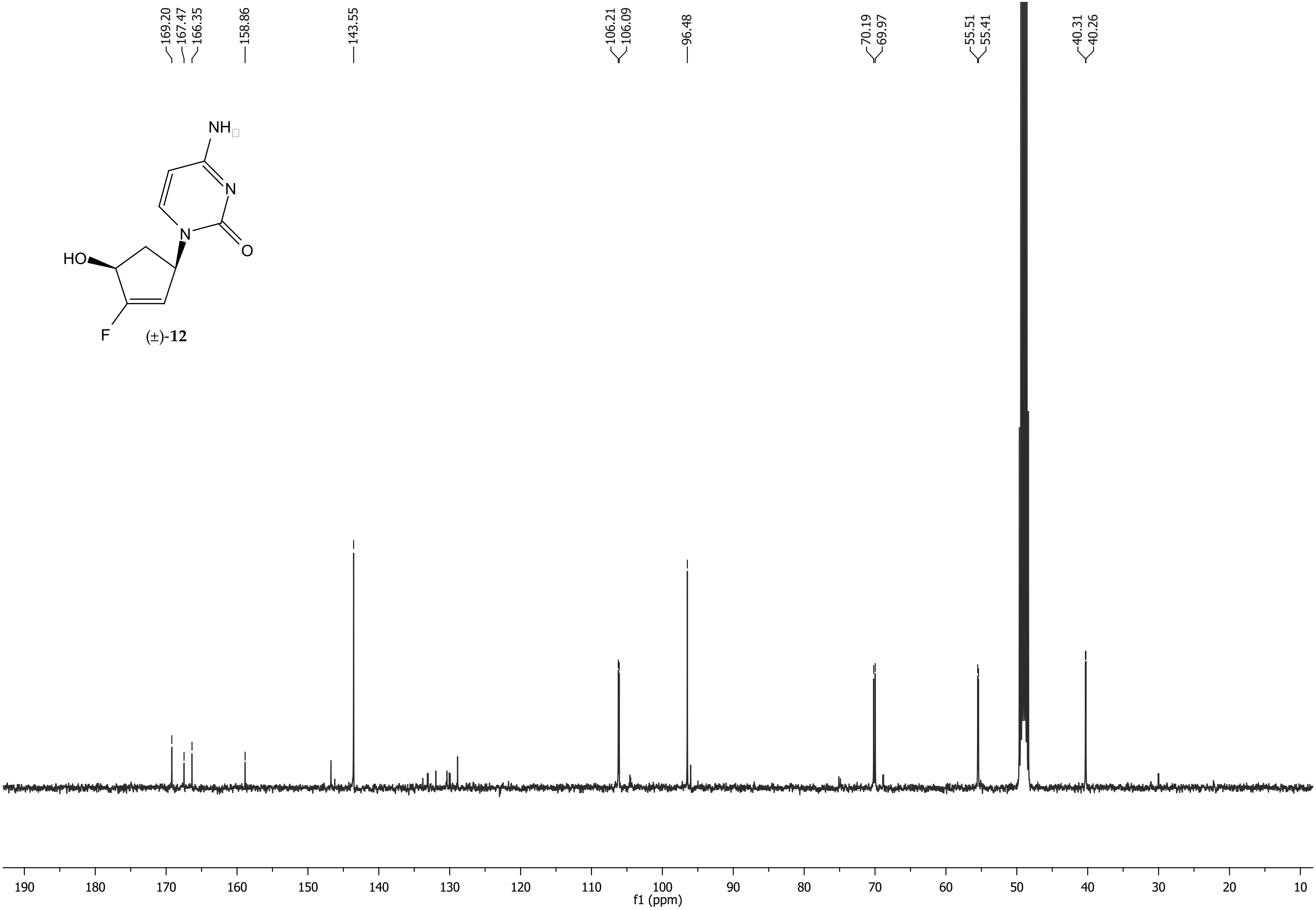

169.20
167.47
166.35
158.86
143.55
106.21
106.09
96.48
70.19
69.97
55.51
55.41
40.31
40.26
NH
N
N
O
HO
F
(±)-12
190
180
170
160
150
140
130
120
110
100
90
80
70
60
50
40
30
20
10
f1 (ppm)

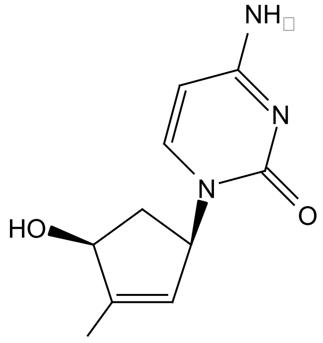
NH
N
N
O
HO
F

(±)-**12**

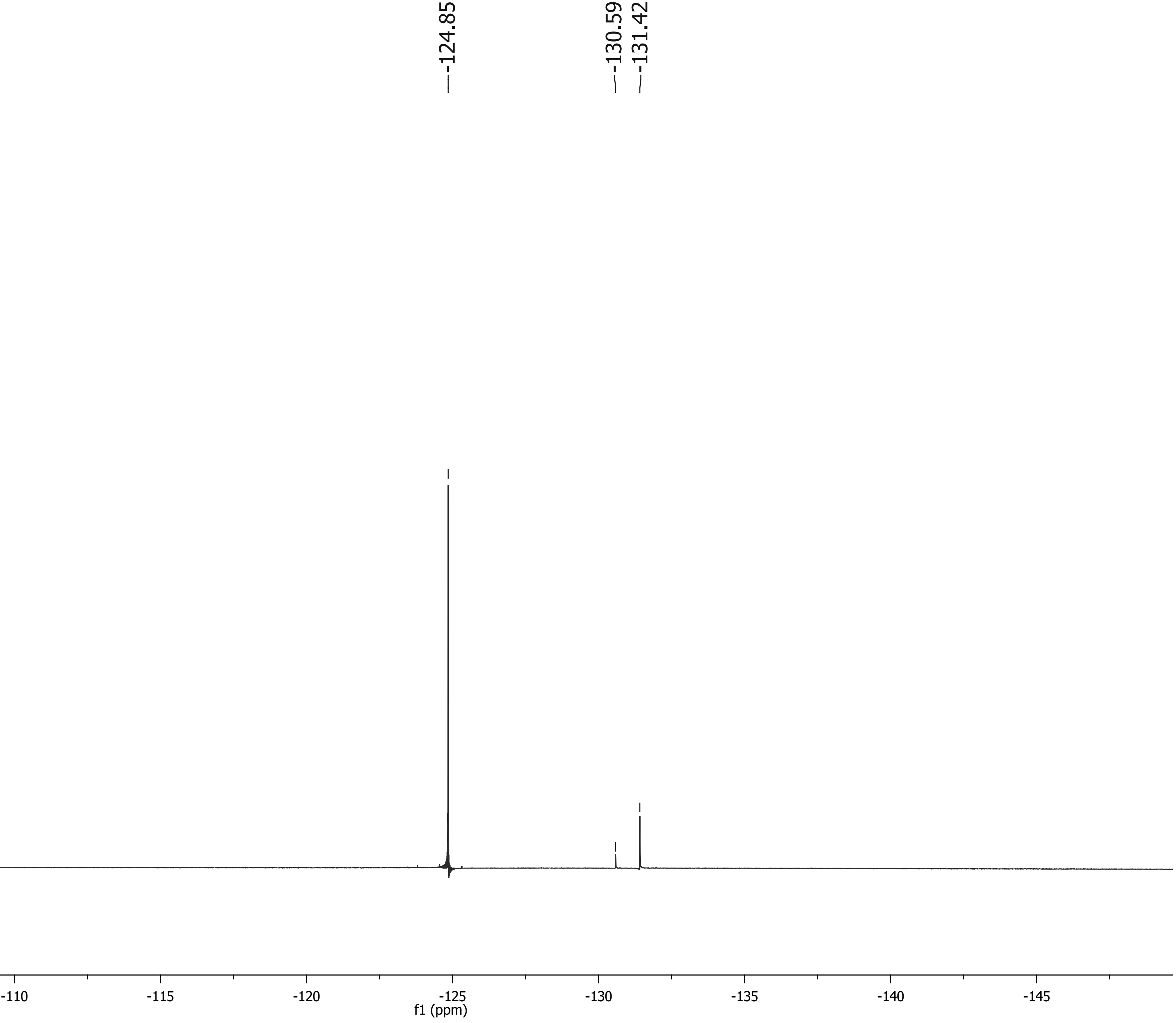
-124.85
-130.59
-131.42
-100
-105
-110
-115
-120
-125
-130
-135
-140
-145
f1 (ppm)

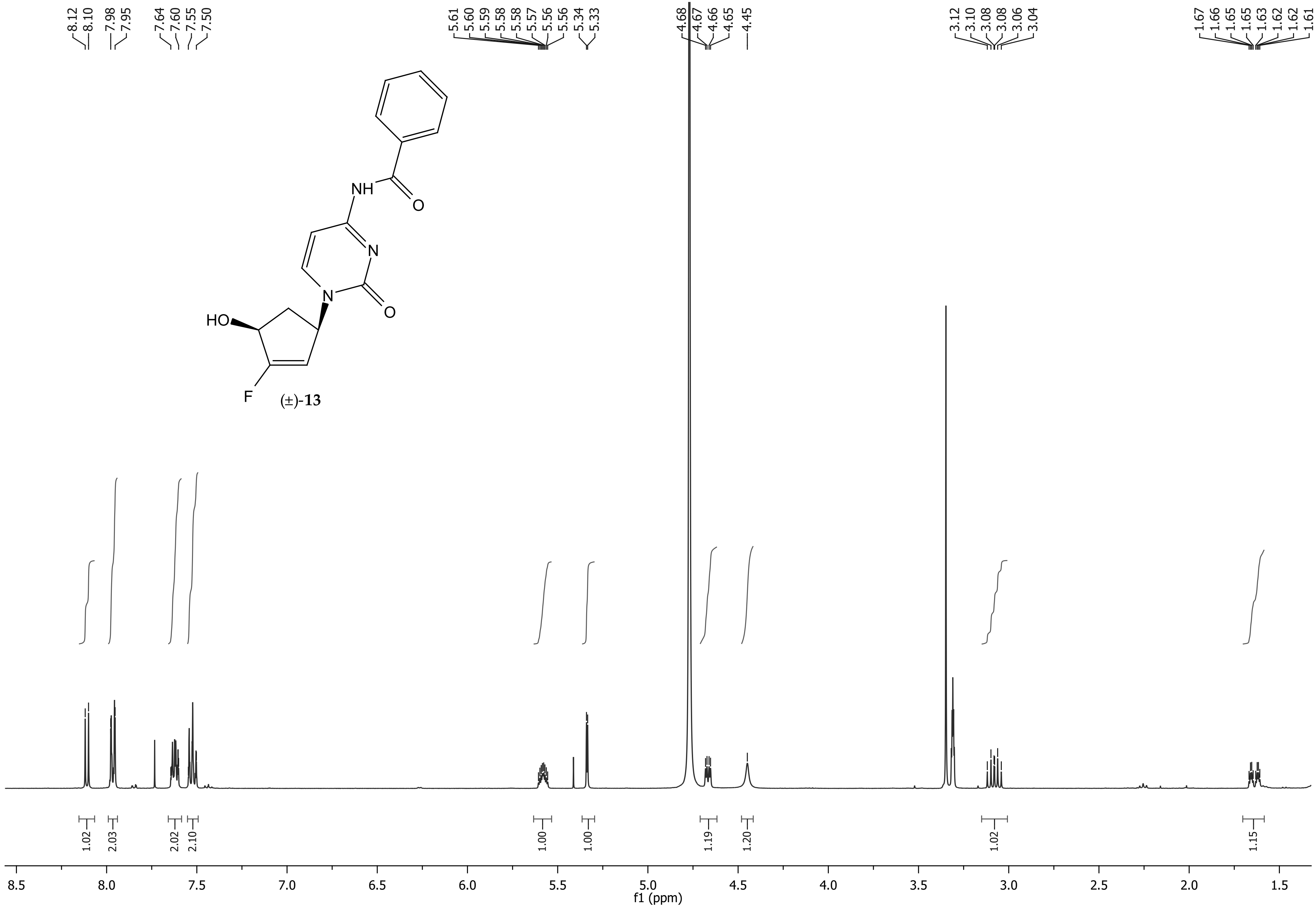
8.12
8.10
7.98
7.95
7.64
7.60
7.55
7.50
5.61
5.60
5.59
5.58
5.58
5.57
5.56
5.56
5.34
5.33
4.68
4.67
4.66
4.65
4.45
3.12
3.10
3.08
3.08
3.06
3.04
1.67
1.66
1.65
1.65
1.63
1.62
1.62
1.61
NH
O
N
N
O
HO
F
(±)-13
1.02
2.03
2.02
2.10
1.00
1.00
1.19
1.20
1.02
1.15
8.5
8.0
7.5
7.0
6.5
6.0
5.5
5.0
4.5
4.0
3.5
3.0
2.5
2.0
1.5
f1 (ppm)

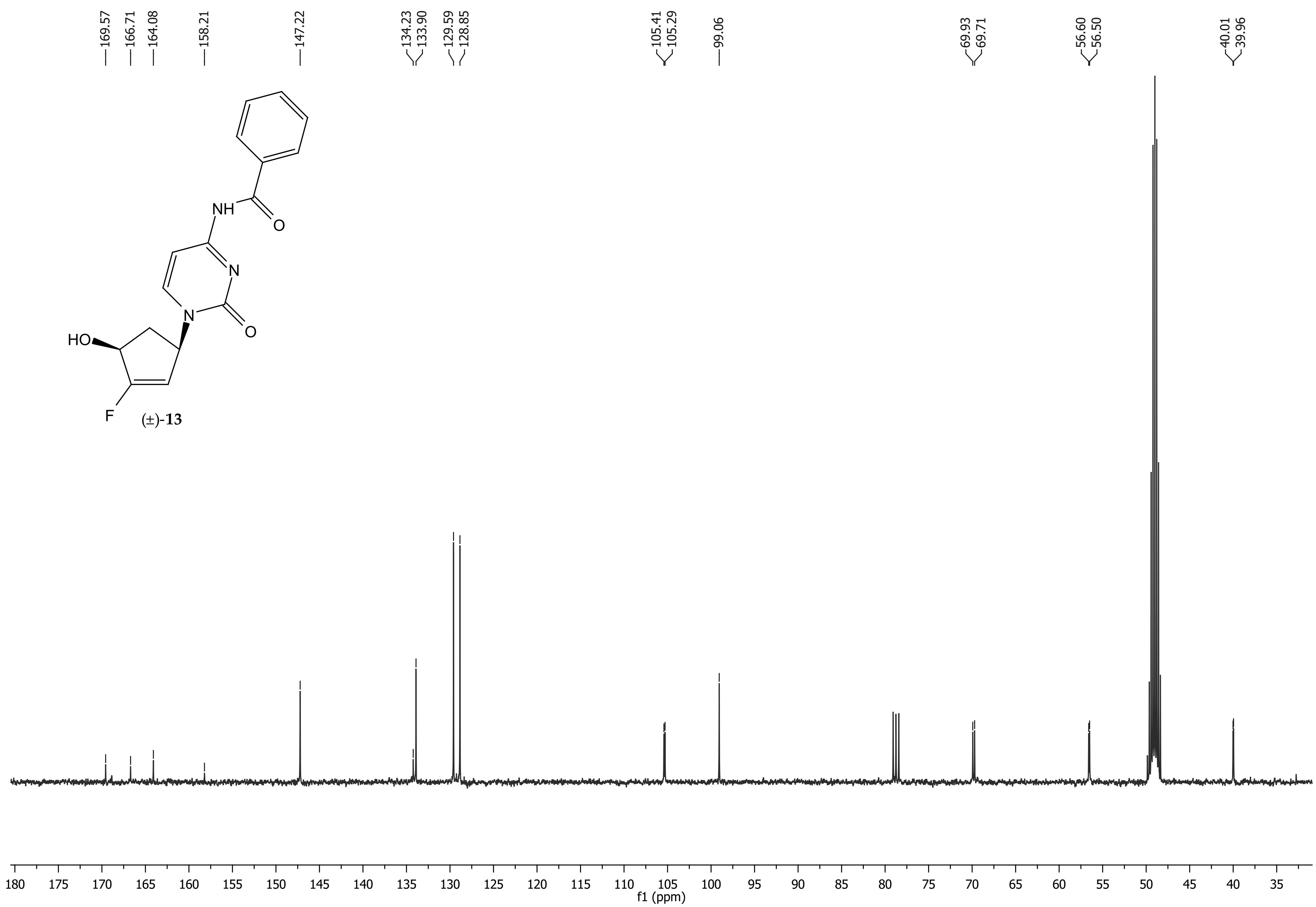
169.57
166.71
164.08
158.21
147.22
134.23
133.90
129.59
128.85
105.41
105.29
99.06
69.93
69.71
56.60
56.50
40.01
39.96
NH
O
N
N
O
HO
F
(±)-13
180 175 170 165 160 155 150 145 140 135 130 125 120 115 110 105 100 95 90 85 80 75 70 65 60 55 50 45 40 35
f1 (ppm)

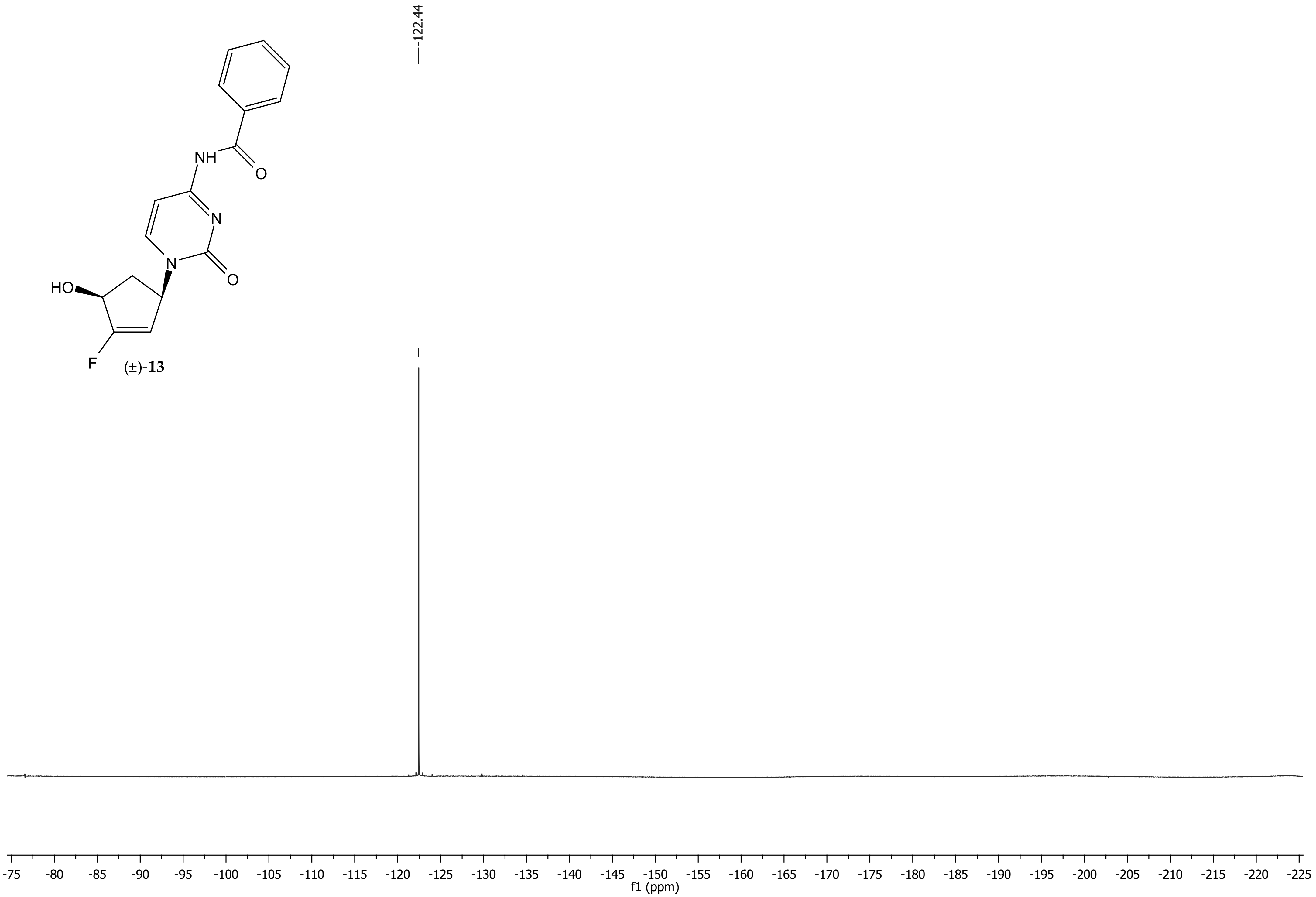
-122.44
NH
O
N
N
O
HO
F
(±)-13
-75 -80 -85 -90 -95 -100 -105 -110 -115 -120 -125 -130 -135 -140 -145 -150 -155 -160 -165 -170 -175 -180 -185 -190 -195 -200 -205 -210 -215 -220 -225
f1 (ppm)

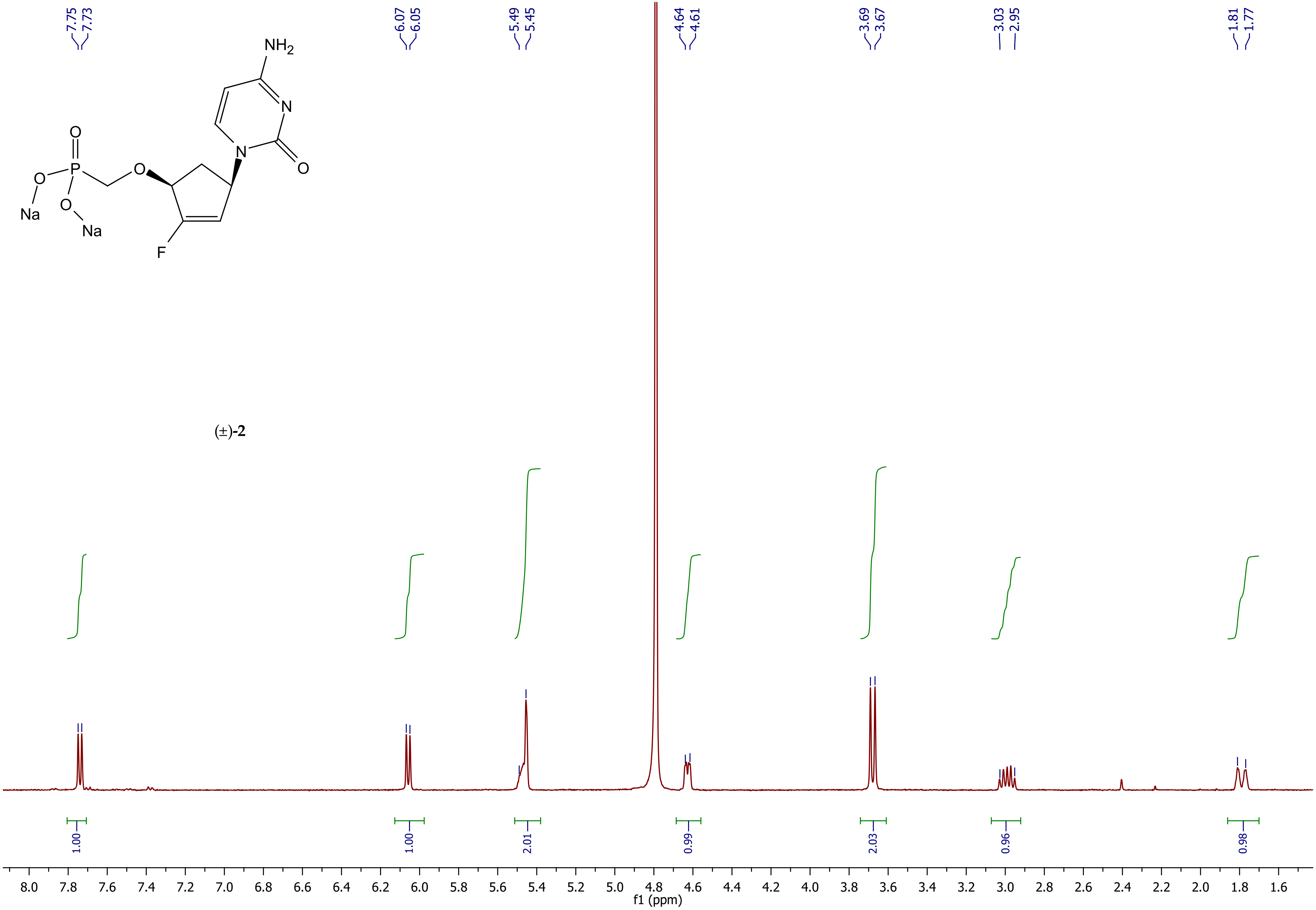
7.75
7.73
6.07
6.05
5.49
5.45
4.64
4.61
3.69
3.67
3.03
2.95
1.81
1.77
NH2
N
O
N
O
P
O
O
Na
O
Na
F
1.00
1.00
2.01
0.99
2.03
0.96
0.98
8.0 7.8 7.6 7.4 7.2 7.0 6.8 6.6 6.4 6.2 6.0 5.8 5.6 5.4 5.2 5.0 4.8 4.6 4.4 4.2 4.0 3.8 3.6 3.4 3.2 3.0 2.8 2.6 2.4 2.2 2.0 1.8 1.6
f1 (ppm)

(±)-**2**

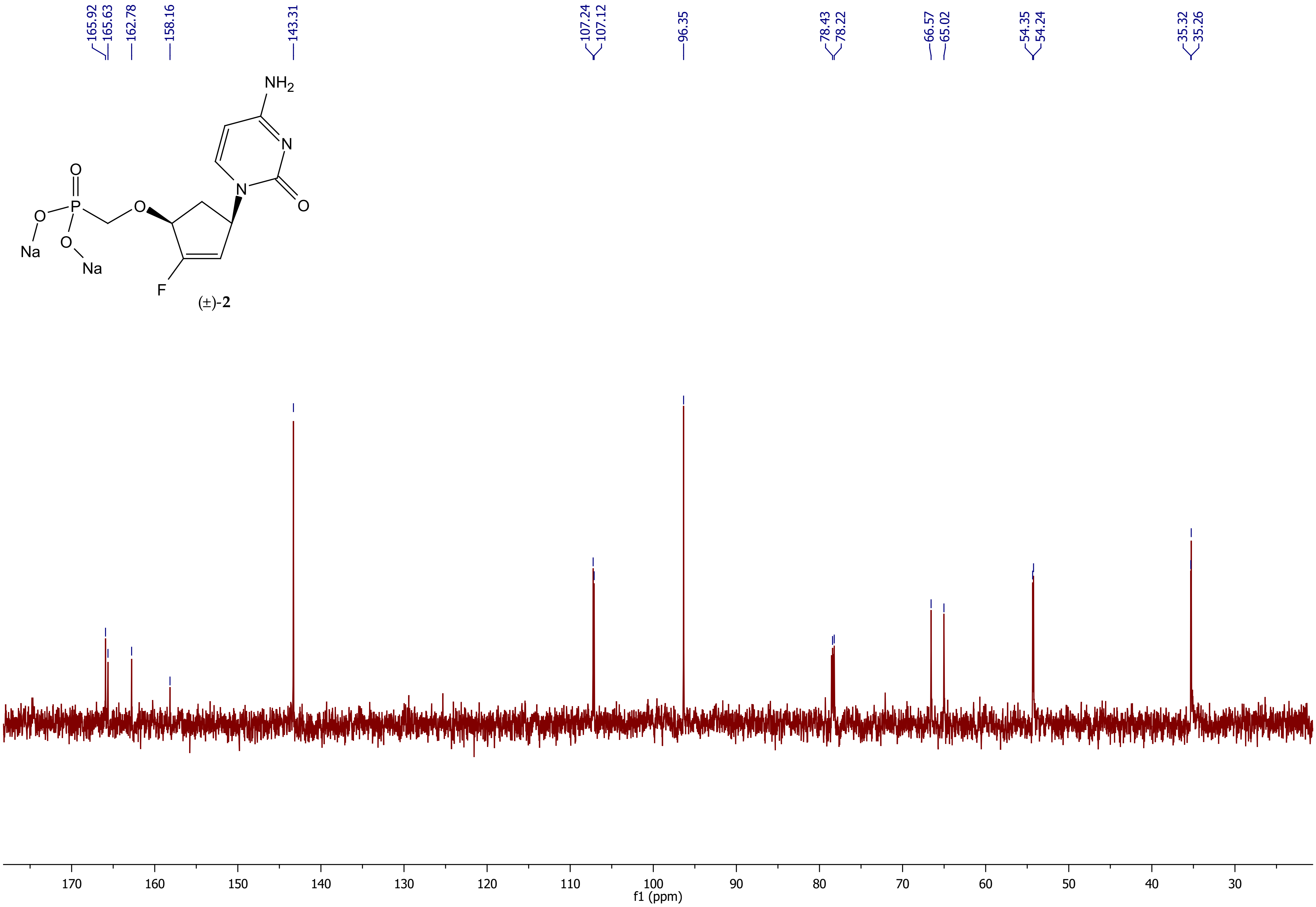
165.92
165.63
162.78
158.16
143.31
107.24
107.12
96.35
78.43
78.22
66.57
65.02
54.35
54.24
35.32
35.26
NH2
N
O
N
O
P
O
O
O
Na
Na
F
(±)-2
170
160
150
140
130
120
110
100
90
80
70
60
50
40
30
f1 (ppm)

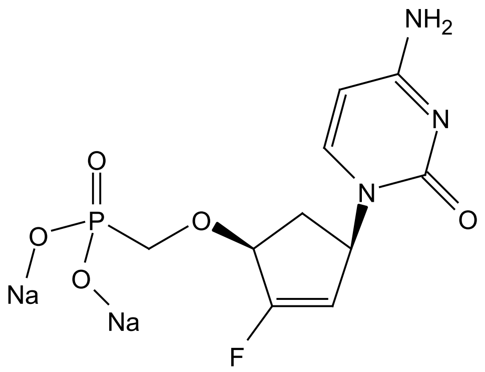
NH2
N
O
N
O
P
O
O
Na
O
Na
F

(±)-**2**

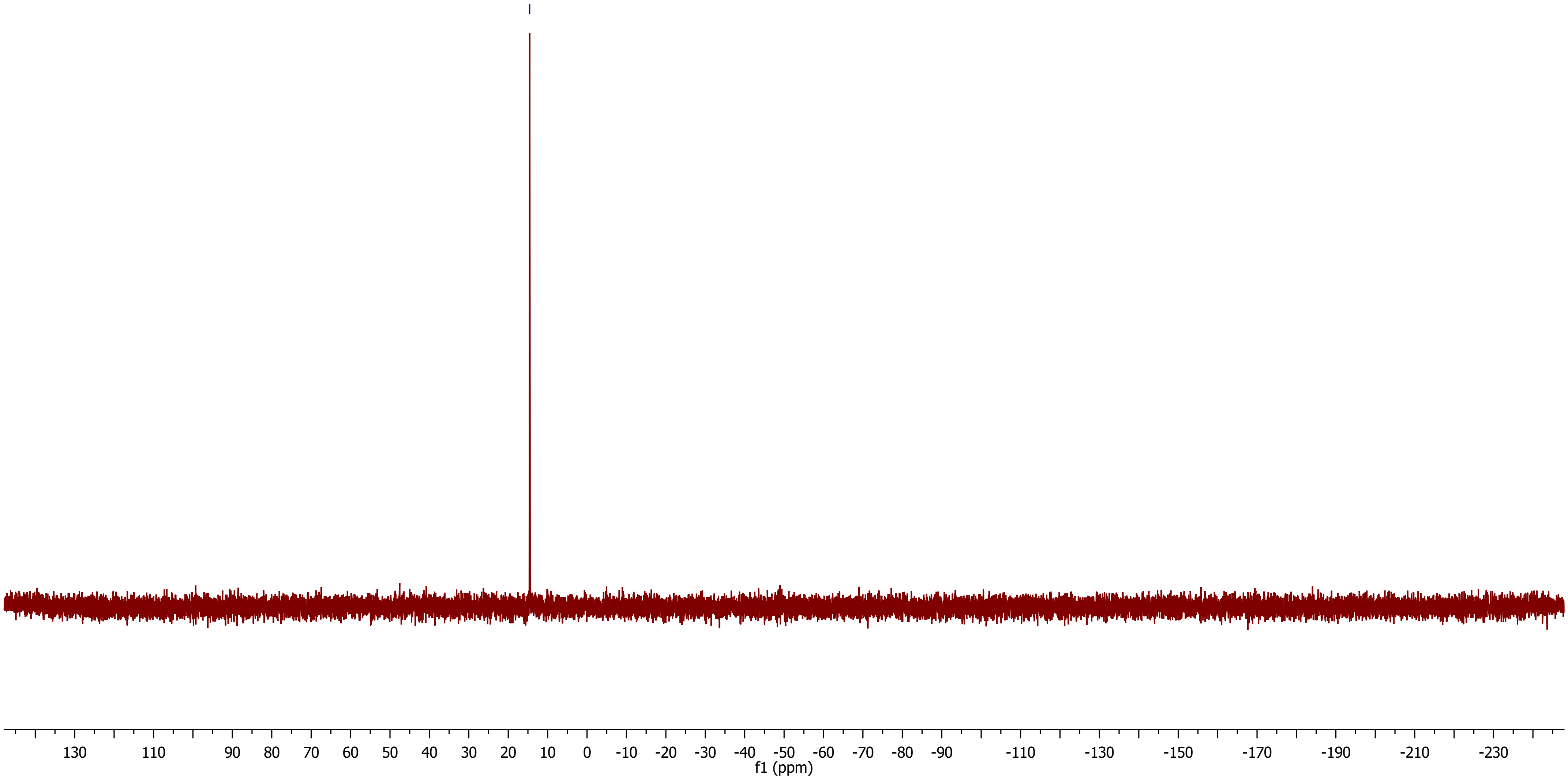
14.55
130
110
90
80
70
60
50
40
30
20
10
0
-10
-20
-30
-40
-50
-60
-70
-80
-90
-110
-130
-150
-170
-190
-210
-230
f1 (ppm)

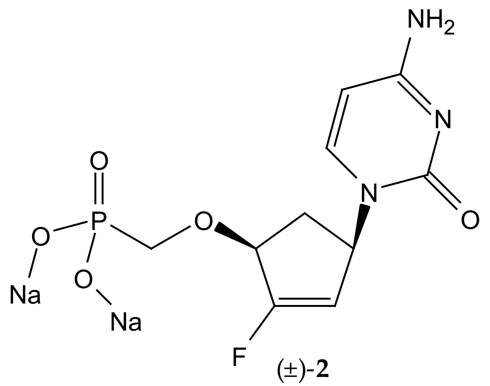
NH2
N
O
N
O
P
O
O
O
Na
Na
F
(±)-**2**

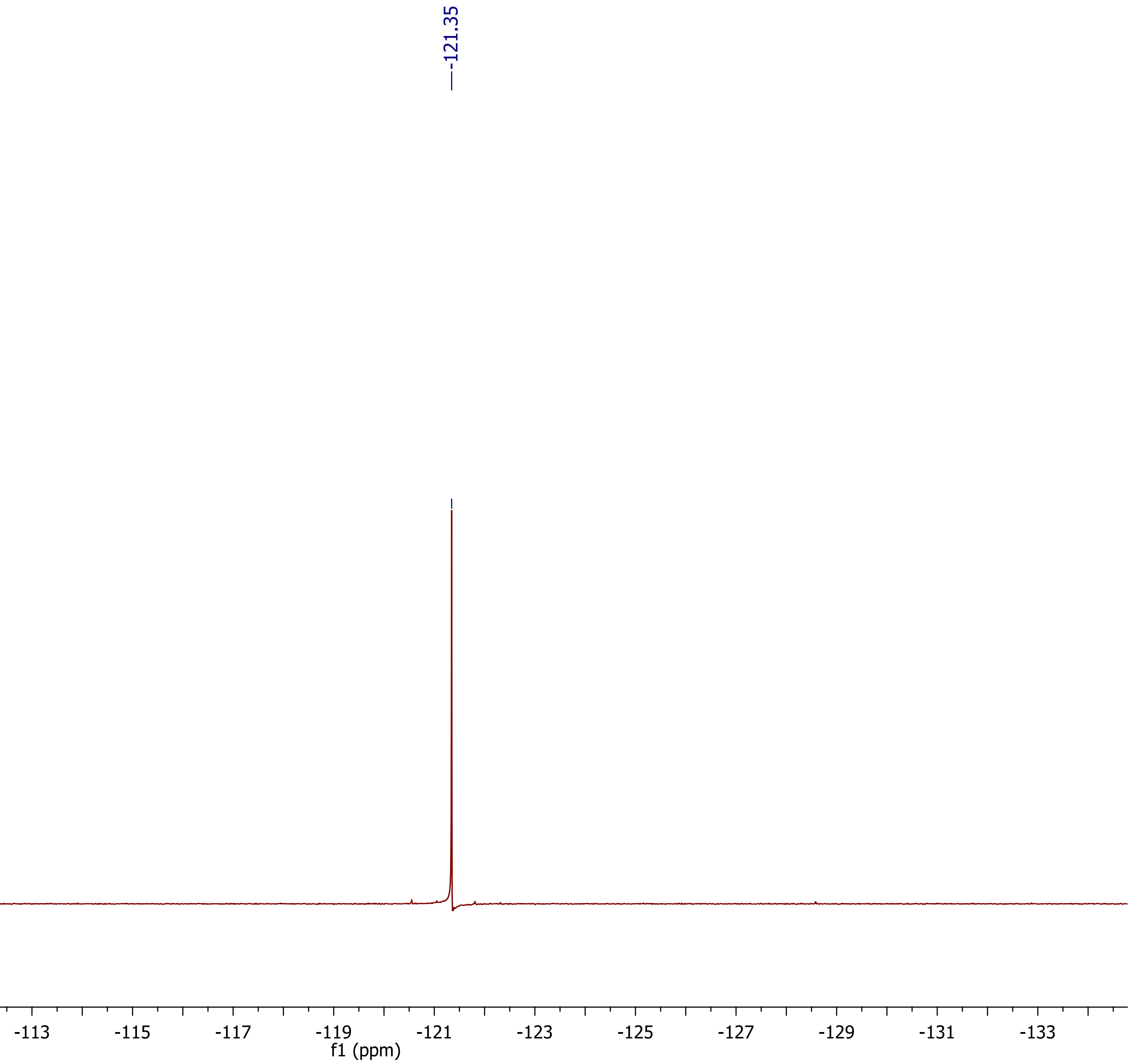
-121.35
-105
-107
-109
-111
-113
-115
-117
-119
-121
-123
-125
-127
-129
-131
-133
f1 (ppm)
